# The relationship between fat talk and body image in Chinese female nursing students: The mediating role of self-acceptance

**DOI:** 10.1371/journal.pone.0351756

**Published:** 2026-07-22

**Authors:** Fengxia Wang, Yuxiao Wang, Yufan Wang, Qihao Yang, Ju Qiu, Jinyan Li, Meng Liu, Xiaowei Wang, Li Li, Xichao Xia

**Affiliations:** Pingdingshan University, Pingdingshan, Henan, China; Universidad Santiago de Cali, COLOMBIA

## Abstract

**Objective:**

This study examined whether self-acceptance mediates the relationship between fat talk and body image among Chinese female nursing students, a group under high appearance pressure.

**Methods:**

A cross-sectional survey was conducted among 1,280 female nursing students from five medical universities in Henan Province, China, Participants were recruited from randomly selected classes, and questionnaires were distributed in person via QR codes.. Participants completed validated scales measuring fat talk (Fat Talk Scale), self-acceptance (Self-Acceptance Questionnaire), and body image (Body Image States Scale). Data were analyzed using correlation and mediation analysis (PROCESS macro, Model 4) with bootstrapping.

**Results:**

Fat talk was positively correlated with both self-acceptance (r = 0.543, p < 0.001) and body image (r = 0.344, p < 0.001). Self-acceptance was also positively correlated with body image (r = 0.457, p < 0.001). After controlling for “relationship status, grade, only child status, and BMI,Mediation analysis revealed a significant indirect effect of fat talk on body image through self-acceptance (effect = 0.174, 95% CI [0.132, 0.222]), accounting for 57.80% of the total effect (total effect = 0.301). The direct effect remained significant (effect = 0.127, 95% CI [0.032, 0.222]).The standardized indirect effect (β = 0.080) indicates a small-to-moderate effect size.

**Conclusion:**

Among Chinese female nursing students, engaging in fat talk conversations is associated with a more positive body image. Self-acceptance accounting for 57.80% of the total effect, though the standardized indirect effect (β = 0.080) suggests a small-to-moderate effect size. This finding highlights the importance of cultural and contextual factors in shaping body discourse and suggests that fostering supportive communication environments and self-acceptance could be beneficial in this population.

## Introduction

Body image, defined as one’s multifaceted perception of, and attitudes toward, their own body [1], is a critical psychological construct strongly linked to mental health outcomes in young women, including disordered eating, depression, and diminished quality of life [2]. For female nursing students, body image concerns may be particularly acute. The professional environment—characterized by standardized uniforms, constant peer comparison, and a clinical focus on physical health—can amplify body surveillance and internalized weight stigma [3,4], making this population a high-risk group worthy of focused investigation.

A common social behavior implicated in body image is “fat talk”—conversations where individuals voice concerns about body size or weight. In Western, individualistic contexts, such talk is predominantly linked to heightened body dissatisfaction, social comparison, and negative affect [5,6]. However, its function may differ in collectivist cultures like China. Emerging evidence suggests that in contexts emphasizing social harmony and interdependence, discussing body concerns may serve as a way to seek and receive social support, strengthen in-group bonds, or normalize shared experiences, potentially mitigating negative outcomes [7,8].It is important to note that this interpretation—that fat talk may be perceived as supportive—is a theoretical speculation, as the perceived valence of such talk was not directly measured in this study. An alternative explanation is that the Chinese version of the Fat Talk Scale may capture more supportive or normalizing weight-related conversations rather than negative self-deprecating talk. This culturally nuanced perspective indicates that the impact of weight-related talk is not universal but is likely filtered through sociocultural and individual psychological processes.

Self-acceptance—the unconditional acknowledgment of oneself, including one’s body—is a key protective factor against appearance-based pressures [9]. Higher self-acceptance predicts lower subsequent body dissatisfaction [10] and buffers against negative media exposure [11]. We posit that in the context of weight-related talk among Chinese female nursing students, if such conversations are perceived as supportive or normative rather than critical, they might bolster rather than undermine self-acceptance. Enhanced self-acceptance, in turn, could lead to a more positive body image. Thus, self-acceptance may serve as a positive psychological pathway, explaining how certain types of weight-related talk relate to adaptive body image outcomes.

Despite this theoretical rationale, the mediating role of self-acceptance in the relationship between fat talk and body image remains untested, especially within culturally distinct and professionally vulnerable groups like Chinese female nursing students. This population exists at a unique intersection: they are embedded in a collectivist culture that traditionally emphasizes modesty and harmony, yet they are increasingly exposed to globalized Western thin-ideals via digital media [12]. Understanding the mechanisms at play for them is essential for developing culturally attuned interventions.

This study aims to investigate this culturally situated mechanism in a sample of Chinese female nursing students. We hypothesize that:

H1: Among Chinese female nursing students, fat talk will be positively associated with body image. (This hypothesis is based on preliminary cultural observations and contrasts with dominant Western literature.)

H2: Self-acceptance will mediate the relationship between fat talk and body image.This study aims to empirically test this hypothesis.

## Methods

### Participants

Female nursing students from five undergraduate medical schools in Henan Province, China, were recruited using a cluster random sampling method (random selection of classes) between July and August 2024.Classes were randomly selected from each university’s roster of nursing classes. QR codes containing the questionnaire were distributed in person during class time to ensure that only students from the selected classes participated. A total of 1,398 students were present in the selected classes and invited to participate; 1,398 questionnaires were distributed, and 1,280 valid questionnaires were completed and returned, yielding a response rate of 91.6%. No missing data were present in the final sample after excluding 118 questionnaires due to incomplete responses or patterned answering (e.g., straight-lining). The average age of the study subjects was (20.49 ± 1.75) years old, and the body mass index (BMI) was (20.12 ± 2.56) kg/m2, of which 288 (22.5%) were in the freshman year, 266 (20.8%) in the sophomore year, 264 (20.6%) in the junior year, and 462 (36.1%) in the senior year, 330 (25.8%) were only children, and 950 (74.2%) were not only children, 548 (42.8%) were in a relationship and 732 (57.2%) were not in a relationship.Ethical approval for the study was granted by the Institutional Review Board of Pingdingshan University (Approval No: 20250317), and informed consent was obtained from all participants,the consent process was implemented in written electronic form.

### Research tools

The Body Image States Scale (BISS) developed by Cash [13] and others was used. The scale consists of six items and is scored on a scale of 1–9, ranging from “extremely dissatisfied” to “extremely satisfied”, Scores were treated as continuous variables in all analyses Higher scores on the scale indicate a higher level of satisfaction with one’s body and a more positive attitude toward body imagery. A total score of ≥25 indicates a “positive body image,” while a total score of <25 indicates a “negative body image” (Cash et al., 2005); these cut-offs are reported for descriptive purposes only and were not used in the statistical analyses. In this study, Cronbach’s α for the full sample was 0.89.

The self-acceptance questionnaire developed by Cong Zhong and Gao Wenfeng [14] was used.This is the standard Chinese version of the scale, which has been widely validated in Chinese populations. The questionnaire consisted of 16 entries. It was rated on a scale of 1–4, from “very much the same” to “very much the opposite”, with a total score of 64. Scores were treated as continuous variables. A higher score indicates a higher level of acceptance. In this study, Cronbach’s α for the full sample was 0.86.

The Fat Talk Scale developed by Clarke [15] was used. The Chinese version of the Fat Talk Scale was produced using a forward-backward translation procedure by two bilingual researchers, followed by pilot testing with 30 nursing students to ensure clarity and cultural appropriateness. The scale consists of 12 items scored 1–5. Cronbach‘s α was 0.81. The scale consists of 12 entries and is scored on a scale of 1–5, from “never” to “always”. The total score ranges from 12 to 60, with ≤24 indicating never or rarely, 25–48 indicating sometimes or often, and >48 indicating always [15]; these cut-offs are reported for descriptive purposes only, while scores were treated as continuous in analyses. In this study, Cronbach’s α for the full sample was 0.81.

### Survey method

The questionnaires were converted into QR codes via Questionnaire Star, and distributed on-site during class sessions through the social media platforms WeChat and QQ.

### Data analysis method

SPSS 22.0 was used to investigate the current status of fat talk, self-acceptance and body image among female nursing students. Normality was assessed using skewness and kurtosis, and all variables were within acceptable ranges (±2), justifying the use of Pearson correlations. For group comparisons in Table 1, independent samples t-tests and one-way ANOVA were used. Assumptions of linear regression (normality of residuals, homoscedasticity, multicollinearity) were checked and met (VIF < 5). The PROCESS plug-in (Model 4) was used to explore the mediating role of female nursing students’ self-acceptance between their fat talk and body image. Using bootstrapping with 5,000 resamples and bias-corrected 95% confidence intervals.. Both unstandardized (B) and standardized (β) coefficients are reported. An adjusted mediation model was conducted controlling for“relationship status, grade, only child status, and BMI, as these variables showed significant associations in preliminary analyses. A post-hoc sensitivity analysis indicated that with N = 1,280 and α = 0.05, the study had 80% power to detect a small indirect effect (f² ≥ 0.02). A p-value < 0.05 indicated statistical significance.

**Table 1:** Differences in fat talk, self-acceptance and body image of nursing students with different demographic variables (scores, x ± s).

factor		n	fat talk	self-acceptance	body image
Only one child	yes	330	36.736 ± 5.843	32.572 ± 2.585	36.227 ± 6.389
	no	950	36.658 ± 5.961	32.470 ± 2.362	36.300 ± 6.427
	*T(1278)*		0.207	0.661	0.177
	*P*		0.836	0.509	0.859
*Cohen’s d*			0.01	0.04	0.01
**Relationship status**	yes	548	39.091 ± 4.511	32.927 ± 2.732	38.591 ± 5.082
	no	732	34.628 ± 2.811	32.174 ± 2.103	34.551 ± 6.759
	*T(1278)*		20.361	5.564	11.726
	*P*		p < 0.001	p < 0.001	p < 0.001
*Cohen’s d*			0.27	0.31	0.66
Grade	freshman	288	37.836 ± 6.489	32.510 ± 2.545	36.542 ± 6.419
	sophomore	266	35.954 ± 5.468	32.398 ± 2.374	36.195 ± 6.228
	Junior	264	36.443 ± 6.097	32.511 ± 2.454	36.462 ± 6.465
	Senior	462	36.506 ± 5.630	32.536 ± 2.354	36.064 ± 6.502
	*F(3,1276)*		0.610	0.487	0.496
	*P*		0.609	0.692	0.685
*η²*			0.001	0.001	0.001

## Results

### Comparison of differences in fat talk, self-acceptance, and body image among female nursing students across demographic variables

There was no difference in the comparison of scores for fat talk, self-acceptance and body image between nursing students who were only children or not and those in different grades (P > 0.05), and there was a difference in the comparison of scores for obesity talk, self-acceptance and body image between nursing students in different relationship statuses (P < 0.05). Cohen’s d is reported as a measure of effect size for significant comparisons. Partial eta-squared (η²) is reported for ANOVA comparisons. No correction for multiple comparisons was applied, as these were exploratory analyses,as shown in Table 1.

### Correlation of fat talk, self-acceptance and body image among female nursing students

There was a significant positive correlation between fat talk and self-acceptance (r = 0.543, p < 0.001) and body image (r = 0.344, p < 0.001), and a significant positive correlation between self-acceptance and body image (r = 0.457, p < 0.001). As shown in Table 2, BMI was not significantly correlated with fat talk (r = 0.015, p = 0.591), self-acceptance (r = −0.022, p = 0.432), or body image (r = −0.018, p = 0.522),as shown in Table 2.

**Table 2:** Correlation analysis of fat talk, self-acceptance and body image among nursing students (*r*).

item	score	fat talk	self-acceptance	body image	
fat talk	36.678 ± 5.928	1			
self-acceptance	32.496 ± 2.420	0.543^**^	1		
body image	36.281 ± 6.415	0.344^**^	0.457^**^	1	
BMI	20.12 ± 2.56	0.015	−0.022	−0.018	1

Note: ** indicates *P* < 0.01.

### The effect of female nursing students’ fat talk on body image: The mediating role of self-acceptance

As can be seen in Table 3, there is a significant positive correlation between the variables, in order to explore the mediating role of self-acceptance of female nursing students between fat talk and body image, the Bootstrap method proposed by Hayes [16]was used to conduct the study, choosing 5000 as the sample size, and indicating a significant mediating effect if the 95% confidence interval does not contain zero., Model 4 was chosen for this study, in which fat talk was used as the independent variable, body image as the dependent variable, and self-acceptance as the mediator variable.Relationship status, grade, Only one child and BMI were included as covariates in the adjusted model.

**Table 3:** Results of regression analysis of female nursing students’ fat talk, body image and self-acceptance.

	body image					self-acceptance					body image				
	*B*	SE	*t*	*p*	*β*	*B*	SE	*t*	*p*	*β*	*B*	SE	*t*	*p*	*β*
Constant	30.124**	4.593	6.559	p < 0.001	–	26.455**	1.770	14.949	p < 0.001	–	11.350*	4.791	2.369	0.018	–
Relationship status	−4.092**	0.405	−10.102	p < 0.001	−0.299	−2.165**	0.156	−13.870	p < 0.001	−0.358	−2.556**	0.418	−6.111	p < 0.001	−0.187
Only child	0.044	0.391	0.111	0.911	0.003	−0.153	0.151	−1.017	0.309	−0.022	0.152	0.377	0.405	0.686	0.010
grade	0.030	0.147	0.205	0.838	0.005	0.070	0.057	1.240	0.215	0.027	−0.020	0.141	−0.140	0.889	−0.003
BMI	0.066	0.203	0.323	0.747	0.008	0.026	0.078	0.329	0.743	0.007	0.047	0.196	0.242	0.809	0.006
fat talk	0.301**	0.047	6.383	p < 0.001	0.189	0.245**	0.018	13.492	p < 0.001	0.348	0.127**	0.048	2.619	0.009	0.080
self-acceptance											0.710**	0.070	10.143	p < 0.001	0.314
R ^2^	0.184					0.380					0.245				
Adjusted R²	0.181					0.377					0.241				
F-value	*F* (5,1274)=57.462,*p* < 0.001					*F* (5,1274)=155.953,*p* < 0.001					*F* (6,1273)=68.863,*p* < 0.001				

* *p* < 0.05 ** *p* < 0.01.

**Fig 1:**
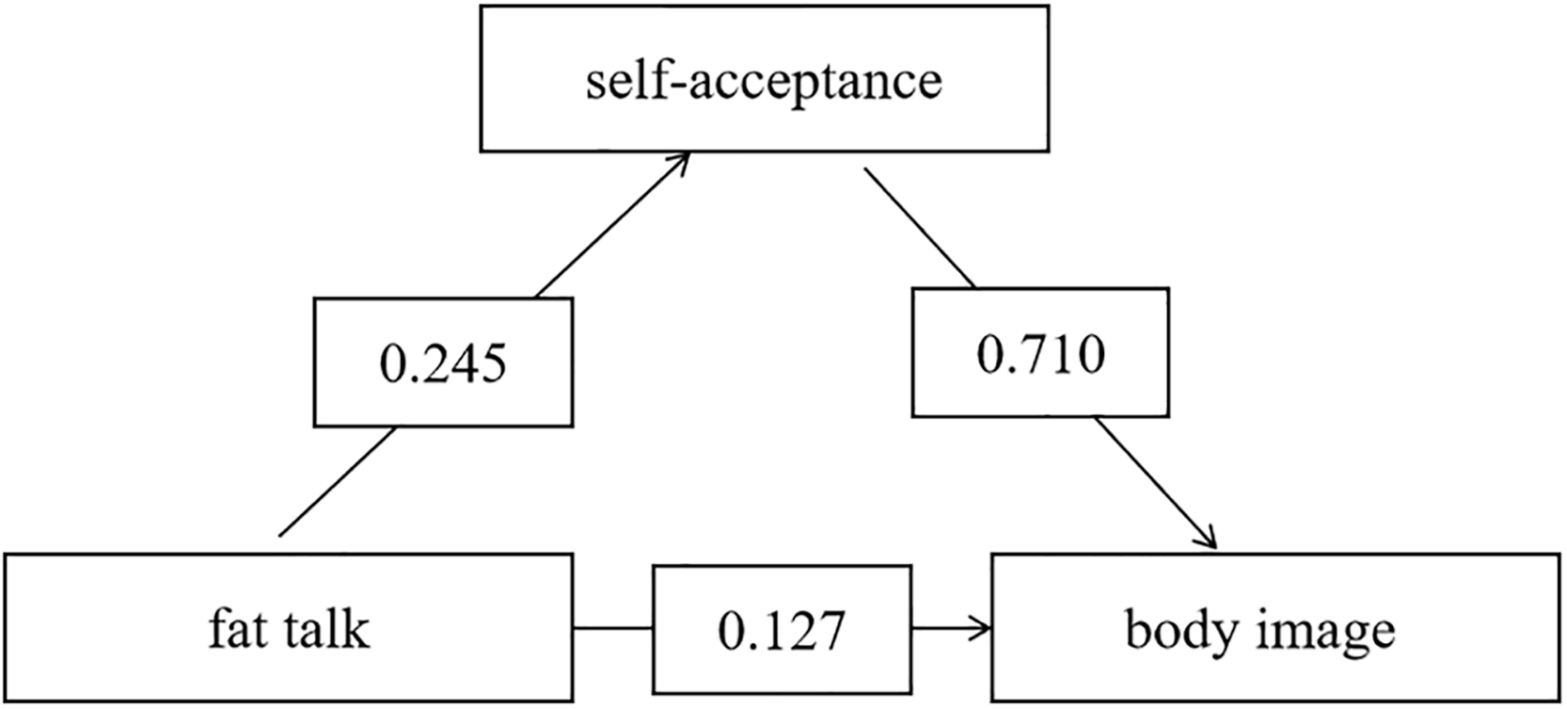
The mediating model of self-acceptance between fat talk and body image in female nursing students.

As can be seen in Table 4,the results indicated that fat talk significantly predicted body image (B = 0.301, p < 0.001). When self-acceptance was introduced as a mediator, the direct effect of fat talk on body image remained significant (B = 0.127, p = 0.009). Mediation analysis revealed a significant indirect effect of fat talk on body image through self-acceptance (effect = 0.174, 95% CI [0.132, 0.222]), accounting for 57.80% of the total effect (total effect = 0.301, 95% CI [0.208, 0.393]), as shown in Table 4.The model illustrating the relationship between fat talk, self-acceptance, and body image among female nursing students is presented in Fig 1.

**Table 4:** Decomposition of mediating effects of female nursing students’ fat talk, body image, and self-acceptance.

Path	Symbol	Meaning	Effect	95% CI	*p*	Proportion of the mediating effect
fat talk=>self-acceptance=>body image	a*b	Indirect effect	0.174	0.132-0.222	p < 0.001	57.80%
fat talk=>self-acceptance	a	X=>M	0.245	0.209-0.280	p < 0.001	
self-acceptance=>body image	b	M=>Y	0.710	0.572-0.847	p < 0.001	
fat talk=>body image	c’	Direct effect	0.127	0.032-0.222	0.009	
fat talk=>body image	c	Total effect	0.301	0.208-0.393	p < 0.001	

## Discussion

This study aimed to explore the relationships between fat talk, self-acceptance, and body image among female nursing students. The results revealed that: (1) Relationship status showed significant differences in scores for fat talk, self-acceptance, and body image, whereas being an only child and grade level did not have significant effects; (2) Fat talk, self-acceptance, and body image were all significantly positively correlated with each other; (3) Self-acceptance played a partial mediating role in the relationship between fat talk and body image.These findings provide new empirical evidence for understanding the internal mechanisms underlying the formation of body image among female nursing students.

### The influence of demographic variables on fat talk, self-acceptance, and body image

This study found that relationship status had significant differences in fat talk, body image, and self-acceptance (*P* < 0.001), with students in romantic relationships scoring higher on each variable. This result aligns with recent research suggesting that intimate relationships may enhance an individual’s acceptance of their own body by providing emotional support and social validation [17]. For example, research has found that a partner’s positive feedback can buffer the negative impact of social physique anxiety on women’s body dissatisfaction [18]. In contrast, being an only child and grade level did not have significant effects. This may imply that, within the nursing student population, family structure characteristics and academic stage are not core factors influencing body image and self-acceptance. This result is consistent with the view of González [19] that body image concerns are widespread among female populations and are more strongly associated with social interpersonal interactions and psychological processes than by demographic background alone.Effect size analyses further supported these findings. For relationship status, the effect sizes ranged from small to medium, with body image showing the largest effect (Cohen’s d = 0.66), indicating that the difference in body image between students in relationships and those not in relationships was not only statistically significant but also practically meaningful. In contrast, the effect sizes for only child status (Cohen’s d = 0.01–0.04) and grade level (Partial η² = 0.001) were very small, suggesting that even if these variables had reached statistical significance, their practical importance would be limited.“

### Correlational relationships between fat talk, self-acceptance, and body image

Correlational analyses showed that fat talk was significantly positively correlated with self-acceptance (*r* = 0.543) and body image (*r* = 0.344), and self-acceptance was also positively correlated with body image (*r* = 0.457).This positive association between fat talk and body image is counterintuitive from the perspective of Western literature, which typically finds negative associations [6]. Several explanations are possible: First, the Chinese version of the Fat Talk Scale may capture more supportive or normalizing weight-related conversations rather than negative self-deprecating talk. Second, in collectivist cultural contexts, discussing body concerns may serve as a way to seek social support and strengthen in-group bonds, potentially mitigating negative outcomes.Third, social desirability bias may have influenced responses, as participants may underreport negative body talk. Fourth, the sample consists of nursing students who may have different health-related discourses compared to the general population. It is important to emphasize that these interpretations are theoretical speculations, as the perceived valence of fat talk was not directly measured. This indicates that, among nursing students, open discussion of topics related to obesity may be associated with more positive body image and higher levels of self-acceptance. This echoes the recent “body positivity” research approach, which suggests that encouraging individuals to discuss body and weight issues in a healthier, more inclusive manner can help reduce internalized stigma and enhance self-worth [20]. However, the correlation strengths were low to moderate, suggesting that fat talk may influence body image through more complex psychosocial mechanisms rather than directly determining it.

### The mediating role of self-acceptance in the relationship between fat talk and body image

Mediation analysis further revealed the internal pathway through which fat talk influences body image. Regression analysis showed that fat talk was positively associated with body image (B = 0.301, P < 0.001). After adding self-acceptance, the direct association remained significant (B = 0.127, P = 0.009), and the mediating effect of self-acceptance was significant (effect = 0.174, 95% CI [0.132, 0.222]), with the mediating effect accounting for 57.80% of the total effect. This suggests that fat talk not only directly affects body image but also indirectly improves it by partially enhancing the level of self-acceptance.This finding aligns with the recent trend emphasizing the application of “self-compassion” and Acceptance and Commitment Therapy (ACT) in body image interventions. Research indicates that self-acceptance can help individuals reduce emotional reactivity to negative body evaluations, thereby promoting a more flexible body image [21]. Furthermore, fat talk, as a socio-cognitive process, may enhance an individual’s acceptance of their own body by promoting cognitive restructuring and emotional expression, thus improving body image [22]. Although the mediating effect in this study was significant, accounted for a substantial proportion (57.80%) of the total effect, the standardized indirect effect (β = 0.080) suggests a small to moderate effect size, indicating that other important mediating or moderating variables might exist.

### Research implications and limitations

This study is the first to verify the mediating role of self-acceptance in the relationship between fat talk and body image among female nursing students, providing new insights for psychological education interventions targeting this group. As future healthcare providers, nursing students’ own body image and self-acceptance levels not only affect their mental health but may also indirectly influence their caregiving attitudes and quality towards patients [23,24]. Therefore, educational programs could incorporate body image enhancement modules based on acceptance and mindfulness, encouraging students to explore body and weight issues in a safe and supportive environment.

This study also has several limitations:

First, the cross-sectional design precludes causal inferences. All findings should be interpreted as associational rather than causal, and the direction of effects cannot be determined. Longitudinal or experimental studies are needed to establish causality.

Second, the sampling procedure, while using random cluster selection, was geographically restricted to five universities in Henan Province, China. The sample is not nationally representative, and generalization to other regions or populations should be made with caution.

Third, the study relied exclusively on self-report measures, which may be subject to social desirability bias and common method bias. Future research could incorporate peer reports, behavioral observations, or implicit measures.

Fourth, key covariates such as relationship status, age, and BMI were included in the adjusted model, but other potentially important variables (e.g., media exposure, peer influences, perfectionism, disordered eating behaviors) were not assessed. These should be considered in future research.

Fifth, although the Chinese version of the Fat Talk Scale showed acceptable reliability, it has not been formally validated in published peer-reviewed studies. The positive associations observed may partly reflect cultural differences in how ‘fat talk’ is conceptualized and reported. Future research should prioritize the psychometric validation of this scale in Chinese populations

## Conclusion

In conclusion, this study demonstrates significant positive correlations among fat talk, self-acceptance, and body image in female nursing students. Relationship status significantly influences these variables, while being an only child and grade level do not. More importantly, self-acceptance partially mediates the relationship between fat talk and body image**..**These findings suggest that fostering open discussions about body-related topics and promoting self-acceptance could be beneficial pathways for improving body image in this population. Future interventions aimed at enhancing the mental well-being of female nursing students may consider incorporating strategies that encourage healthy communication about body image and cultivate self-acceptance, while also addressing the potential influence of relationship contexts. Further research is needed to explore additional mediating factors and to establish causal relationships.
