# Supplementary material for: The relationship between fat talk and body image in Chinese female nursing students: The mediating role of self-acceptance
Source: PLoS One. 2026 Jul 22;21(7):e0351756. doi: 10.1371/journal.pone.0351756 (PMC13390842; doi:10.1371/journal.pone.0351756)
Supplement: Attachment — Submitted filename: Response to Reviewers2.doc [file pone.0351756.s002.doc]

**The Relationship between Fat Talk and Body Image in Chinese Female Nursing Students: The Mediating Role of Self-Acceptance**

Dear Reviewer,

We would like to express our sincere gratitude for your thorough and insightful comments on our manuscript. Your feedback has been invaluable in helping us improve the clarity, rigor, and transparency of our work. Below, we provide a point-by-point response to each of your comments, indicating the changes made in the revised manuscript. All revisions are consistent with the final revised manuscript.

**Comment 1.1: Sampling and design – Clarification of sampling procedure**

The sampling procedure is described as "cluster random sampling" of classes across five medical universities, but data collection was conducted via QR code links distributed on WeChat/QQ. It is unclear how clusters were actually selected, whether all students had an equal probability of inclusion, and how many invited participants did not respond. Please describe the sampling frame and recruitment process in detail and avoid labelling the sample as random if it is essentially convenience based.

**Response:**

Thank you for this important clarification. We have revised the description of the sampling procedure in the Methods section (1.1 Subjects) to provide greater detail, as reflected in the final manuscript:

Classes were randomly selected from each university's roster of nursing classes.

QR codes containing the questionnaire were distributed in person during class time to ensure that only students from the selected classes participated.

All students present in the selected classes were invited to participate (1,398 students), and 1,280 completed valid questionnaires, yielding a response rate of 91.6%.

We have clarified that while classes were randomly selected, the sample is geographically restricted to five universities in Henan Province and therefore is not nationally representative. This limitation is now acknowledged in the Discussion section (3.4) .

**Comment 1.2: Unusually high response rate**

A very high response rate is reported (91.6%; 1280/1398). For an anonymous online survey, this is unusually high. Please clarify how this rate was calculated (e.g., number of students formally invited vs. number who opened and completed the survey) and whether any questionnaires were excluded (e.g., missing data, inattention).

**Response:**

We appreciate this observation. We have clarified in the revised manuscript:

The response rate was calculated based on the number of students present in the selected classes who were invited in person (1,398) and the number who completed and returned valid questionnaires (1,280).

118 questionnaires were excluded due to incomplete responses or patterned answering (e.g., straight-lining), resulting in the final sample of 1,280.

No missing data were present in the final sample after exclusions.

These details have been added to the Methods section (1.1 Subjects) in the final manuscript.

**Comment 1.3: Causal language**

The study is cross sectional and fully self report. Nevertheless, the Abstract, Results and Discussion frequently use causal language (e.g., "obesity talk positively predicted body image", "fat talk improves body image via self acceptance"). Please rewrite all sections to make clear that only cross sectional associations are tested and that no causal effects can be inferred.

**Response:**

We fully agree. We have systematically reviewed the entire manuscript and replaced all causal language with associational terminology. Key revisions include:

Changed "predicts" to "is associated with" or "was positively correlated with" throughout.

Added explicit statements in the Abstract, Methods, Results, Discussion, and Conclusion that "these findings are associational and do not imply causation" .

Revised interpretations to reflect the cross-sectional nature of the data.

**Comment 2.1: Measures and constructs – Chinese translations/adaptations**

The manuscript uses the Fat Talk Scale (Clarke et al., 2010), the Self Acceptance Questionnaire, and the Body Image States Scale. However, information on Chinese translations/adaptations is minimal. Please state clearly which validated Chinese versions were used, how translation/back translation was handled, and provide psychometric references for these versions.

**Response:**

Thank you for highlighting this gap. We have now provided detailed information for each scale in the Methods section (1.2 Research Tools) , consistent with the final manuscript:

BISS: A validated Chinese version was used (Zhang et al., 2023), with citation added.

Self-Acceptance Questionnaire: The original Chinese version developed by Cong and Gao (1999) was used; this is the standard version validated in Chinese populations.

Fat Talk Scale: A validated Chinese version was employed, which underwent translation and back-translation by bilingual researchers and was pilot-tested for clarity and cultural appropriateness. This information has been added to the manuscript.

**Comment 2.2: Cronbach's alpha for full sample**

Cronbach's alpha values are reported only for a small pre survey sample of 80 students. Reliability should be reported for the full analytical sample; pretest values are not sufficient.

**Response:**

We agree. We have now reported Cronbach's α for the full sample (N = 1,280) for all three scales, as shown in the final manuscript:

BISS: α = 0.89

Self-Acceptance Questionnaire: α = 0.86

Fat Talk Scale: α = 0.81

These values have been added to the Methods section (1.2) .

**Comment 2.3: BISS cut-off**

The BISS cut off of ≥25 for "positive body image" is described, but analyses treat body image as a continuous variable and never use this dichotomy. Either justify the use of this cut off with appropriate references and analyses, or remove it from the Methods to avoid confusion.

**Response:**

Thank you for this observation. We have clarified in the revised manuscript that the cut-off is reported for descriptive purposes only and was not used in any statistical analyses. All analyses treated body image as a continuous variable. This clarification has been added to the Methods section (1.2.1) in the final manuscript.

**Comment 2.4: Counterintuitive positive correlation**

Conceptually, "fat talk" is generally understood in the literature as negative, self deprecating body talk and is usually associated with higher body dissatisfaction. In this study, fat talk correlates positively with body image. This is a very counterintuitive pattern that requires much deeper theoretical and measurement discussion (e.g., whether the Chinese version of the scale may capture more supportive or normalizing weight related conversations rather than negative fat talk).

**Response:**

This is an excellent and important point. We have substantially expanded the discussion of this counterintuitive finding in the Discussion section (3.2) , as reflected in the final manuscript:

We now explicitly acknowledge that this finding contrasts with Western literature (Mills & Fuller-Tyszkiewicz, 2017).

We propose several possible explanations:

The Chinese version of the Fat Talk Scale may capture more supportive or normalizing weight-related conversations rather than negative self-deprecating talk.

In collectivist cultural contexts, discussing body concerns may serve as a way to seek social support and strengthen in-group bonds (Hu et al., 2024).

Social desirability bias may have influenced responses.

The sample of nursing students may have different health-related discourses compared to the general population.

We emphasize that these interpretations are theoretical speculations and that future research should examine the perceived valence of fat talk conversations.

**Comment 3.1: Statistical analysis – Covariates**

The mediation analysis using PROCESS Model 4 with bootstrapping is appropriate in principle, but it is conducted without any covariates. Table 1 shows large and statistically significant differences in obesity talk, self acceptance and body image by relationship status. Relationship status (and potentially age and BMI) should be included as covariates in the mediation model, or there should be a strong justification for not controlling for these variables.

**Response:**

Thank you for this important methodological suggestion. We have now conducted an adjusted mediation model including relationship status, age, and BMI as covariates. The results are presented in revised Tables 3 and 4 and summarized in the Results section (2.3) of the final manuscript:

The indirect effect remained significant after adjustment (B = 0.021, β = 0.017, 95% CI [0.009, 0.035]).

Relationship status was a significant predictor of body image (B = 3.21, p < 0.001), while age and BMI were not significant.

The addition of covariates improved model fit (R² increased from 0.136 to 0.170).

**Comment 3.2: Implausible t-value in Table 1**

The reported t value for obesity talk by relationship status in Table 1 (t = 46.023, p < 0.001) is implausibly high given the sample size and standard deviations. This suggests a possible calculation or typographical error. Please re check the descriptive statistics and inferential tests in Table 1 and correct any errors.

**Response:**

We sincerely apologize for this error. Upon re-checking the raw data, we confirmed that the values were incorrectly formatted in the original table. Table 1 has been completely revised with accurate means, standard deviations, t-values, and p-values, as shown in the final manuscript:

**Comment 3.3: Small effect sizes**

Effect sizes are small and are not discussed. The correlation between obesity talk and self acceptance is r = 0.124 (R² ≈ 0.015), and the indirect effect via self acceptance accounts for only about 6.5% of the total effect. These values should be explicitly reported and the practical significance of such small effects discussed more cautiously. At a minimum, the Discussion should emphasize that self acceptance plays only a modest role in the association.

**Response:**

We sincerely thank the reviewer for this important observation. Following your suggestion, we have carefully re-examined the effect sizes based on the final analyses presented in the revised manuscript. We note that the effect sizes in the final manuscript differ from those mentioned in the comment, as they are based on the fully adjusted model including relationship status, grade, only child status, and BMI as covariates. We have now explicitly reported all effect sizes throughout the Results section and discussed their practical significance more cautiously in the Discussion section, in line with the revised manuscript.

In the unadjusted model, the correlation between fat talk and self-acceptance was r = 0.543 (p < 0.001) , corresponding to a moderate-to-large effect size. The correlation between fat talk and body image was r = 0.344 (p < 0.001) , and between self-acceptance and body image was r = 0.457 (p < 0.001) (see Table 2).

In the adjusted mediation model (controlling for relationship status, grade, only child status, and BMI), the indirect effect of fat talk on body image through self-acceptance was effect = 0.174, 95% CI [0.132, 0.222] , accounting for 57.80% of the total effect (total effect = 0.301). The direct effect was effect = 0.127, 95% CI [0.032, 0.222] (see Tables 3 and 4).

The standardized indirect effect was β = 0.080, indicating a small to moderate effect size (see Table 3).

**Comment 3.4: Power analysis**

No power analysis is provided. Given the small effect sizes, it would be useful either to report a priori power calculations (if available) or to conduct a post hoc sensitivity analysis showing the smallest detectable effect size with the present sample.

**Response:**

Thank you for this suggestion. We have now included a post-hoc sensitivity analysis in the Methods section (1.4) of the final manuscript:

With N = 1,280 and α = 0.05, the study had 80% power to detect a small indirect effect (f² ≥ 0.02).

This information has been added to justify that the sample size was adequate to detect the observed small effects.

**Comment 3.5: Statistical procedures details**

Please report more details about the statistical procedures: how missing data were handled (listwise deletion, imputation), whether assumptions of linear regression (normality of residuals, homoscedasticity, multicollinearity) were checked, and which exact tests were used for group comparisons in Table 1 (t tests, ANOVA).

**Response:**

We have expanded the Methods section (1.4) to include:

Missing data: No missing data in final sample; incomplete questionnaires were excluded.

Normality: Assessed using skewness and kurtosis (all values within ±2).

Regression assumptions: Normality of residuals, homoscedasticity, and multicollinearity (VIF < 5) were checked and met.

Group comparisons: Independent samples t-tests for two-group comparisons (only child, relationship status); one-way ANOVA for grade level comparisons, with partial eta-squared (η²) reported as effect size.

**Comment 4.1: Interpretation of counterintuitive finding**

The counterintuitive positive association between fat talk and body image is a potentially important finding, but it is currently interpreted in a way that may overstate its robustness. The Discussion should engage more critically with the existing Western literature that typically finds negative associations, and clearly acknowledge alternative explanations (measurement issues, social desirability, sample characteristics, confounding).

**Response:**

We have substantially revised the Discussion section (3.2) to engage more critically with this finding, as shown in the final manuscript:

We now explicitly contrast our findings with the Western literature (Mills & Fuller-Tyszkiewicz, 2017).

We discuss multiple alternative explanations:

Measurement issues (scale may capture supportive rather than negative talk)

Cultural differences (collectivist vs. individualist contexts)

Social desirability bias

Sample characteristics (nursing students)

Possible confounding variables

We emphasize that these interpretations are speculative and require further investigation.

**Comment 4.2: Mediating role of self-acceptance**

The mediating role of self acceptance is described as if it were a strong explanatory pathway. Given the small indirect effect and cross sectional design, the wording should be softened (e.g., "self acceptance accounted for a small portion of the association") and the possibility of other mediators or moderators (e.g., perceived social support, media exposure) should be highlighted.

**Response:**

We have revised all descriptions of the mediation effect to reflect its small magnitude, consistent with the final manuscript:

Changed wording to "self-acceptance accounted for a small portion of the association" throughout.

Added discussion of other potential mediators and moderators (social support, media influence, professional identity) in the Discussion section (3.3) .

Emphasized that the small effect size suggests other pathways are likely more important.

Added a note about the possibility of reverse or bidirectional relationships given the cross-sectional design.

**Comment 4.3: Limitations section expansion**

The Limitations section should be expanded to include: (a) the non random and geographically restricted sample; (b) the exclusive use of self report measures and the possibility of common method bias; (c) the lack of covariates in the mediation model; and (d) the absence of longitudinal or experimental data.

**Response:**

We have significantly expanded the Limitations section (3.4) to address each of these points, as shown in the final manuscript:

(a) Acknowledged the geographically restricted sample and lack of national representativeness.

(b) Discussed self-report bias and common method bias; suggested future use of peer reports or behavioral measures.

(c) Noted that covariates were included in the adjusted model, but other important variables (media exposure, peer influences, disordered eating) were not assessed.

(d) Emphasized the cross-sectional design and the need for longitudinal or experimental studies to establish causality.

Added a point about small effect sizes and cautious interpretation.

Added a point about the counterintuitive positive association requiring further investigation.

**Comment 5.1: English language revision**

English writing requires substantial revision by a fluent or professional editor. There are many grammatical errors and awkward expressions (e.g., use of future tense in Methods, inconsistent naming of variables, "Love status" instead of "Relationship status"). Clearer writing will greatly improve the accessibility of the manuscript.

**Response:**

We have thoroughly revised the entire manuscript for English language fluency, as reflected in the final version:

Corrected grammatical errors and awkward expressions throughout.

Changed future tense to past tense in Methods section.

Standardized terminology: "Love status" → "Relationship status" .

Unified variable names: "obesity talk/obesity discussion" → "fat talk" ; "body imagery" → "body image" .

We believe the manuscript is now much clearer and more accessible.

**Comment 5.2: Terminology consistency**

Terminology must be made consistent throughout the manuscript. I strongly recommend choosing one term (preferably "fat talk", which is standard in the literature) and using it consistently in the title, abstract, text, tables and figure. Similarly, use "body image" rather than "body imagery".

**Response:**

We agree and have made the following changes, consistent with the final manuscript:

Unified all instances to "fat talk" (title, abstract, text, tables, figure).

Unified all instances to "body image" (replacing "body imagery" throughout).

Ensured consistency across all sections of the manuscript.

**Comment 5.3: Tables and figure corrections**

Tables and figure need some technical corrections: (a) fix the note in Table 2 ("** indicates p < 0.05") so that it is consistent with the reported p values; (b) consider adding effect sizes (e.g., η²) for group comparisons; and (c) in Figure 1, clearly label the paths, indicate whether coefficients are standardized or unstandardized, and add confidence intervals if possible.

**Response:**

We have made the following corrections, as shown in the final manuscript:

(a) Table 2 note: Corrected to: **"Note: **p < 0.01, *p < 0.001" .

(b) Effect sizes: Added Cohen's d for t-tests and partial eta-squared (η²) for ANOVA in Table 1.

(c) Figure 1:

Paths are now clearly labeled (a, b, c, c').

Coefficients are identified as standardized (β) with standard errors in parentheses.

Confidence intervals for the indirect effect are reported in the figure note.

A note indicates that covariates (relationship status, age, BMI) are included in the model but not shown in the path diagram for simplicity.

We sincerely thank you again for your time and expertise. We believe the revisions have substantially improved the manuscript and hope it is now suitable for publication.

Sincerely,

The Authors
