# Supplementary material for: The relationship between fat talk and body image in Chinese female nursing students: The mediating role of self-acceptance
Source: PLoS One. 2026 Jul 22;21(7):e0351756. doi: 10.1371/journal.pone.0351756 (PMC13390842; doi:10.1371/journal.pone.0351756)
Supplement: Attachment — Submitted filename: respond to reviewers.doc [file pone.0351756.s004.doc]

**Comment 1**:Data Availability (DOI not working): Please make the Data Availability Statement consistent and fully PLOS-compliant. In your response letter you provide a Figshare DOI (10.6084/m9.figshare.28345678.v1), but when I tried to open it, it returned DOI NOT FOUND. Please verify that the DOI is correct and activated, and that the repository item is publicly accessible without restriction. The manuscript currently still states: "All relevant data are within the paper and its Supporting Information files." This must be updated to the correct working public DOI/URL, and the statement should specify what files are provided (dataset + data dictionary/codebook).

**Our response and revision**:The data that support the findings of this study are openly available in Zenodo at DOI:10.5281/zenodo.20018315.

Accordingly, we have revised the Data Availability Statement at the end of the manuscript .

**Comment 2:**Ethics/IRB approval number inconsistency: Please verify the correct IRB approval number and report it consistently throughout all materials. The response letter / ethics form reports 20250317, while the manuscript Methods reports 20240317.

**Our response and revision:**We thank the reviewer for catching this typographical error. After verifying with the Institutional Review Board of Pingdingshan University, the correct approval number is 20250317. The number in the Methods section has been corrected accordingly.

**Comment 3：**Incomplete sentence about indirect-effect significance: Please complete the sentence describing the mediation significance criterion: it should state that the 95% confidence interval does not include 0 (zero); (currently the sentence is incomplete).

**Our response and revision:**We have completed the sentence in the Results section where the mediation significance criterion is described. The incomplete sentence has been revised to state the full criterion.

Location: Results → Mediation analysis subsection.

Revised text: indicating a significant mediating effect if the 95% confidence interval does not contain zero.

**Comment 4**:Bootstrap: Please correct the bootstrap wording. The manuscript currently refers to "choosing 5,000 as the sample size," which is incorrect. The study sample size is the number of participants included in the analysis (N = 1,280). 5,000 refers to the number of bootstrap resamples used to estimate the confidence interval for the indirect effect. Please revise the wording accordingly (e.g., bootstrapping with 5,000 resamples).

**Our response and revision**:We have corrected the inaccurate wording regarding the bootstrap procedure. The phrase "choosing 5,000 as the sample size" has been replaced with the correct description.

Location: Methods → Data analysis method section.

Original text: choosing 5000 as the sample size

Revised text: bootstrapping with 5,000 resamples

**Comment 5：**p-value: Please do not report p-values as "0.000" (or p<=0.:001). Use p < 0.001 or report exact p-values where available (e.g., p = 0.009), consistently across text and all tables.

**Our response and revision**:We have replaced all instances of p = 0.000 with p < 0.001 throughout the manuscript, including in text and all tables (Tables 1, 3, and 4). Exact p-values (e.g., p = 0.009, p = 0.018) have been retained where appropriate.

Specific revisions:

Table 1: Changed P = 0.000 to p < 0.001 for relationship status comparisons.

Table 4: Changed the p-value in the Path row from 0.000 to < 0.001.

Text: All instances of p = 0.000 have been changed to p < 0.001.

Locations: Throughout the manuscript (Abstract, Results, Tables 1, 3, 4).

**Comment 5:**Table cross-references: Please correct table cross-references. The text currently refers to mediation results "as shown in Table 2," but the mediation decomposition is reported in Table 4.

**Our response and revision**:We have corrected the cross-reference error. The text now correctly points to Table 4 for the mediation decomposition results.

Location: Results → Correlation subsection.

Original text: as shown in Table 2

Revised text: as shown in Table 4

We have also verified that all other table cross-references in the manuscript are correct.

**Comment 6**:Associational language (cross-sectional design): Please replace remaining causal verbs (e.g., predicts, affects, improves, influences) with strictly associational language appropriate for cross-sectional data (e.g., is associated with, is linked to).

**Our response and revision**:We have replaced causal verbs with associational language throughout the manuscript, consistent with the cross-sectional design of our study.

Locations: Abstract, Discussion (first and third subsections), and throughout the manuscript where causal verbs appeared.

**Comment 7**:Terminology consistency: Please use one main term consistently (preferably fat talk, as in the title and scale name). The manuscript still switches between fat talk, obesity talk, and obesity discussion (e.g., Abstract/Methods and section headings). If these are intended as synonyms, please standardize them; if they are intended as distinct constructs, please define them explicitly.

**Our response and revision**:We agree with the reviewer that terminology should be consistent. We have standardized all terms to fat talk, as this matches the title, the scale name (Fat Talk Scale), and the dominant terminology in the literature. The terms obesity talk and obesity discussion have been replaced with fat talk throughout the manuscript.

**Specific revisions**:

All other instances of obesity talk and obesity discussion in the manuscript have been similarly changed to fat talk. These are intended as synonyms, and we have now standardized to a single term.

Locations: Abstract (Methods), Results (subsection titles and text), and throughout the manuscript.

**Summary of Revisions**

We believe that all remaining issues raised by Reviewer #1 have been fully addressed. The revised manuscript is now publication-ready. We thank the reviewer and the academic editor for their time and constructive feedback, which have significantly improved the quality of our paper.

We look forward to your final decision.
