# Supplementary material for: The relationship between fat talk and body image in Chinese female nursing students: The mediating role of self-acceptance
Source: PLoS One. 2026 Jul 22;21(7):e0351756. doi: 10.1371/journal.pone.0351756 (PMC13390842; doi:10.1371/journal.pone.0351756)
Supplement: Peer review history

# Peer review: Decision Letter 0

**DOI:** 10.1371/journal.pone.0351756.r001

**Authors:** Alejandro Botero Carvajal

2 Mar 2026

PONE-D-25-68898

The Relationship between Obesity Talk and Body Image in Chinese Female Nursing Students: The Mediating Role of  Self-Acceptance

PLOS One

Dear Dr. Xia,

Thank you for submitting your manuscript to PLOS ONE. After careful consideration, we feel that it has merit but does not fully meet PLOS ONE’s publication criteria as it currently stands. Therefore, we invite you to submit a revised version of the manuscript that addresses the points raised during the review process.

Please submit your revised manuscript by Apr 16 2026 11:59PM. If you will need more time than this to complete your revisions, please reply to this message or contact the journal office at plosone@plos.org. When you're ready to submit your revision, log on to https://www.editorialmanager.com/pone/ and select the 'Submissions Needing Revision' folder to locate your manuscript file.

Please include the following items when submitting your revised manuscript:

- A letter that responds to each point raised by the academic editor and reviewer(s). You should upload this letter as a separate file labeled 'Response to Reviewers'.
- A marked-up copy of your manuscript that highlights changes made to the original version. You should upload this as a separate file labeled 'Revised Manuscript with Track Changes'.
- An unmarked version of your revised paper without tracked changes. You should upload this as a separate file labeled 'Manuscript'.

If you would like to make changes to your financial disclosure, please include your updated statement in your cover letter. Guidelines for resubmitting your figure files are available below the reviewer comments at the end of this letter.

If applicable, we recommend that you deposit your laboratory protocols in protocols.io to enhance the reproducibility of your results. Protocols.io assigns your protocol its own identifier (DOI) so that it can be cited independently in the future. For instructions see: https://journals.plos.org/plosone/s/submission-guidelines#loc-laboratory-protocols. Additionally, PLOS ONE offers an option for publishing peer-reviewed Lab Protocol articles, which describe protocols hosted on protocols.io. Read more information on sharing protocols at https://plos.org/protocols?utm_medium=editorial-email&utm_source=authorletters&utm_campaign=protocols.

We look forward to receiving your revised manuscript.

Kind regards,

Alejandro Botero Carvajal, Ph.D

Academic Editor

PLOS One

Journal requirements:

When submitting your revision, we need you to address these additional requirements.

1. Please ensure that your manuscript meets PLOS ONE's style requirements, including those for file naming. The PLOS ONE style templates can be found at

https://journals.plos.org/plosone/s/file?id=wjVg/PLOSOne_formatting_sample_main_body.pdf and

https://journals.plos.org/plosone/s/file?id=ba62/PLOSOne_formatting_sample_title_authors_affiliations.pdf

2. We note that your Data Availability Statement is currently as follows: [All relevant data are within the manuscript and its Supporting Information files.]

Please confirm at this time whether or not your submission contains all raw data required to replicate the results of your study. Authors must share the “minimal data set” for their submission. PLOS defines the minimal data set to consist of the data required to replicate all study findings reported in the article, as well as related metadata and methods (https://journals.plos.org/plosone/s/data-availability#loc-minimal-data-set-definition).

For example, authors should submit the following data:

- The values behind the means, standard deviations and other measures reported;

- The values used to build graphs;

- The points extracted from images for analysis.

Authors do not need to submit their entire data set if only a portion of the data was used in the reported study.

If your submission does not contain these data, please either upload them as Supporting Information files or deposit them to a stable, public repository and provide us with the relevant URLs, DOIs, or accession numbers. For a list of recommended repositories, please see https://journals.plos.org/plosone/s/recommended-repositories.

If there are ethical or legal restrictions on sharing a de-identified data set, please explain them in detail (e.g., data contain potentially sensitive information, data are owned by a third-party organization, etc.) and who has imposed them (e.g., an ethics committee). Please also provide contact information for a data access committee, ethics committee, or other institutional body to which data requests may be sent. If data are owned by a third party, please indicate how others may request data access.

3. PLOS requires an ORCID iD for the corresponding author in Editorial Manager on papers submitted after December 6th, 2016. Please ensure that you have an ORCID iD and that it is validated in Editorial Manager. To do this, go to ‘Update my Information’ (in the upper left-hand corner of the main menu), and click on the Fetch/Validate link next to the ORCID field. This will take you to the ORCID site and allow you to create a new iD or authenticate a pre-existing iD in Editorial Manager.

If the reviewer comments include a recommendation to cite specific previously published works, please review and evaluate these publications to determine whether they are relevant and should be cited. There is no requirement to cite these works unless the editor has indicated otherwise.

[Note: HTML markup is below. Please do not edit.]

Reviewers' comments:

Reviewer's Responses to Questions

**Comments to the Author**

1. Is the manuscript technically sound, and do the data support the conclusions?

The manuscript must describe a technically sound piece of scientific research with data that supports the conclusions. Experiments must have been conducted rigorously, with appropriate controls, replication, and sample sizes. The conclusions must be drawn appropriately based on the data presented.

Reviewer #1: Partly

Reviewer #2: Partly

**********

2. Has the statistical analysis been performed appropriately and rigorously?

Reviewer #1: No

Reviewer #2: Yes

**********

3. Have the authors made all data underlying the findings in their manuscript fully available?

The PLOS Data policy requires authors to make all data underlying the findings described in their manuscript fully available without restriction, with rare exception (please refer to the Data Availability Statement in the manuscript PDF file). The data should be provided as part of the manuscript or its supporting information, or deposited to a public repository. For example, in addition to summary statistics, the data points behind means, medians and variance measures should be available. If there are restrictions on publicly sharing data—e.g. participant privacy or use of data from a third party—those must be specified.

Reviewer #1: No

Reviewer #2: Yes

**********

4. Is the manuscript presented in an intelligible fashion and written in standard English?

PLOS ONE does not copyedit accepted manuscripts, so the language in submitted articles must be clear, correct, and unambiguous. Any typographical or grammatical errors should be corrected at revision, so please note any specific errors here.

Reviewer #1: No

Reviewer #2: No

**********

5. Review Comments to the Author

Please use the space provided to explain your answers to the questions above. You may also include additional comments for the author, including concerns about dual publication, research ethics, or publication ethics. (Please upload your review as an attachment if it exceeds 20,000 characters)

Reviewer #1: The analytic approach fits the aims of the study, but the manuscript requires substantial revision before the results can be considered robust and reproducible.Because the design is cross-sectional, the mediation analysis must be interpreted as associational rather than causal. Several statements (e.g., in the Abstract, Discussion, and Conclusion) currently imply an intervention-oriented perspective that cross-sectional data simply cannot support. These should be significantly toned down.The statistical methods section is far too sparse to replicate the analyses. It is essential to state whether Pearson or Spearman correlations were used and why, and explain how missing data were handled. Regarding the PROCESS analysis, please describe the bootstrap procedure clearly (5,000 bootstrap resamples, not “sample size”) and report what type of confidence interval was used (e.g., percentile vs bias-corrected). Furthermore, clarify whether the reported B coefficients are unstandardized; given that the variables are on different scales, reporting standardized coefficients (β) alongside B is strongly recommended for a meaningful interpretation of effect magnitudes.Table 3/Figure 1 report the a, b, and c′ paths, but please also report uncertainty for each path (SEs and/or 95% CIs) and clarify whether coefficients are unstandardized (B) or standardized (β). This would make the mediation results easier to interpret.

BMI is reported descriptively but does not appear in the correlation table or in the mediation model. The role of BMI and the links between BMI/weight status and obesity talk, self-acceptance, and body image, are not addressed, and this omission introduces a significant risk of confounding. Please report BMI’s bivariate associations with the key variables, and consider an adjusted mediation model (or sensitivity analyses) controlling for BMI and basic demographics (e.g., age, year of study, relationship status, only-child status).Table 1 also needs re-examination: the relationship-status comparisons show very large t-values and values that are hard to reconcile with the other descriptive statistics, which suggests possible data-reporting error or formatting problems. Verify the table and ensure the tests are reported correctly, including degrees of freedom, exact p-values, and effect sizes. Given the number of outcomes and demographic comparisons, I also suggest stating whether any correction for multiple comparisons was applied.

In addition, the footnote under Table 2 (** indicates p<0.05) may be confusing, since "**" is often used for smaller p-values, and elsewhere the manuscript frequently reports p<0.001.

Regarding the instruments of measure, the manuscript lists cut-offs/score ranges for the Fat Talk Scale and the BISS, but it is unclear whether these are validated thresholds and whether they were used analytically or only as descriptive categories. Please provide sources for these thresholds and clarify whether scores were handled as continuous or categorical in the actual analysis. Reliability is reported from a small pre-survey (80), report reliability indices for the full sample (1280) as well. Clarify whether validated Chinese versions of the BISS and Fat Talk Scale were used, and describe translation/adaptation procedures if applicable.

In the Introduction and Discussion, the authors suggest that obesity talk is perceived as supportive or normative, yet perceived valence is not measured. This should be presented as a possible interpretation rather than a mechanism demonstrated by the data. Additionally, given the close links between body image and eating-related symptoms, it would strengthen the limitations to acknowledge that disordered eating was not assessed (e.g., restraint, binge eating, compensatory behaviors), as this could be relevant to interpretation and to future model extensions. The Data Availability Statement is very general.

Finally, the sampling procedure would benefit from clarification. The manuscript describes cluster random sampling, but survey administration is described as distribution via a QR code through WeChat/QQ. Please explain how the sampled clusters were approached and how participation was limited to the selected classes. The manuscript is generally understandable, but the English and statistical phrasing should be edited carefully (there are incomplete sentences and imprecise expressions such as “p<0.05 indicated the difference”), and terminology should be made consistent (obesity talk/obesity discussion; body image/body imagery).

Reviewer #2: The study addresses an interesting and culturally relevant question: whether “fat talk” (here labelled “obesity talk”) is positively associated with body image among Chinese female nursing students, and whether self-acceptance mediates this relationship. The topic is suitable for PLOS ONE, but in its current form the manuscript has several major methodological, statistical and interpretative limitations, in addition to important language issues. I therefore recommend major revision. My main comments are below.

1. Sampling and design

1.1 The sampling procedure is described as “cluster random sampling” of classes across five medical universities, but data collection was conducted via QR code links distributed on WeChat/QQ. It is unclear how clusters were actually selected, whether all students had an equal probability of inclusion, and how many invited participants did not respond. Please describe the sampling frame and recruitment process in detail and avoid labelling the sample as random if it is essentially convenience based.

1.2 A very high response rate is reported (91.6%; 1280/1398). For an anonymous online survey, this is unusually high. Please clarify how this rate was calculated (e.g., number of students formally invited vs. number who opened and completed the survey) and whether any questionnaires were excluded (e.g., missing data, inattention).

1.3 The study is cross sectional and fully self report. Nevertheless, the Abstract, Results and Discussion frequently use causal language (e.g., “obesity talk positively predicted body image”, “fat talk improves body image via self acceptance”). Please rewrite all sections to make clear that only cross sectional associations are tested and that no causal effects can be inferred.

2. Measures and constructs

2.1 The manuscript uses the Fat Talk Scale (Clarke et al., 2010), the Self Acceptance Questionnaire, and the Body Image States Scale. However, information on Chinese translations/adaptations is minimal. Please state clearly which validated Chinese versions were used, how translation/back translation was handled, and provide psychometric references for these versions.

2.2 Cronbach’s alpha values are reported only for a small pre survey sample of 80 students. Reliability should be reported for the full analytical sample; pretest values are not sufficient.

2.3 The BISS cut off of ≥25 for “positive body image” is described, but analyses treat body image as a continuous variable and never use this dichotomy. Either justify the use of this cut off with appropriate references and analyses, or remove it from the Methods to avoid confusion.

2.4 Conceptually, “fat talk” is generally understood in the literature as negative, self deprecating body talk and is usually associated with higher body dissatisfaction. In this study, fat talk correlates positively with body image. This is a very counterintuitive pattern that requires much deeper theoretical and measurement discussion (e.g., whether the Chinese version of the scale may capture more supportive or normalizing weight related conversations rather than negative fat talk).

3. Statistical analysis

3.1 The mediation analysis using PROCESS Model 4 with bootstrapping is appropriate in principle, but it is conducted without any covariates. Table 1 shows large and statistically significant differences in obesity talk, self acceptance and body image by relationship status. Relationship status (and potentially age and BMI) should be included as covariates in the mediation model, or there should be a strong justification for not controlling for these variables.

3.2 The reported t value for obesity talk by relationship status in Table 1 (t = 46.023, p < 0.001) is implausibly high given the sample size and standard deviations. This suggests a possible calculation or typographical error. Please re check the descriptive statistics and inferential tests in Table 1 and correct any errors.

3.3 Effect sizes are small and are not discussed. The correlation between obesity talk and self acceptance is r = 0.124 (R² ≈ 0.015), and the indirect effect via self acceptance accounts for only about 6.5% of the total effect. These values should be explicitly reported and the practical significance of such small effects discussed more cautiously. At a minimum, the Discussion should emphasize that self acceptance plays only a modest role in the association.

3.4 No power analysis is provided. Given the small effect sizes, it would be useful either to report a priori power calculations (if available) or to conduct a post hoc sensitivity analysis showing the smallest detectable effect size with the present sample.

3.5 Please report more details about the statistical procedures: how missing data were handled (listwise deletion, imputation), whether assumptions of linear regression (normality of residuals, homoscedasticity, multicollinearity) were checked, and which exact tests were used for group comparisons in Table 1 (t tests, ANOVA).

4. Interpretation and discussion

4.1 The counterintuitive positive association between fat talk and body image is a potentially important finding, but it is currently interpreted in a way that may overstate its robustness. The Discussion should engage more critically with the existing Western literature that typically finds negative associations, and clearly acknowledge alternative explanations (measurement issues, social desirability, sample characteristics, confounding).

4.2 The mediating role of self acceptance is described as if it were a strong explanatory pathway. Given the small indirect effect and cross sectional design, the wording should be softened (e.g., “self acceptance accounted for a small portion of the association”) and the possibility of other mediators or moderators (e.g., perceived social support, media exposure) should be highlighted.

4.3 The Limitations section should be expanded to include: (a) the non random and geographically restricted sample; (b) the exclusive use of self report measures and the possibility of common method bias; (c) the lack of covariates in the mediation model; and (d) the absence of longitudinal or experimental data.

5. Reporting, language and formatting

5.1 English writing requires substantial revision by a fluent or professional editor. There are many grammatical errors and awkward expressions (e.g., use of future tense in Methods, inconsistent naming of variables, “Love status” instead of “Relationship status”). Clearer writing will greatly improve the accessibility of the manuscript.

5.2 Terminology must be made consistent throughout the manuscript. I strongly recommend choosing one term (preferably “fat talk”, which is standard in the literature) and using it consistently in the title, abstract, text, tables and figure. Similarly, use “body image” rather than “body imagery”.

5.3 Tables and figure need some technical corrections: (a) fix the note in Table 2 (“** indicates p < 0.05”) so that it is consistent with the reported p values; (b) consider adding effect sizes (e.g., η²) for group comparisons; and (c) in Figure 1, clearly label the paths, indicate whether coefficients are standardized or unstandardized, and add confidence intervals if possible.

**********

6. PLOS authors have the option to publish the peer review history of their article (what does this mean?). If published, this will include your full peer review and any attached files.

If you choose “no”, your identity will remain anonymous but your review may still be made public.

**Do you want your identity to be public for this peer review?**  For information about this choice, including consent withdrawal, please see our Privacy Policy.

Reviewer #1: No

Reviewer #2: No

**********

[NOTE: If reviewer comments were submitted as an attachment file, they will be attached to this email and accessible via the submission site. Please log into your account, locate the manuscript record, and check for the action link "View Attachments". If this link does not appear, there are no attachment files.]

To ensure your figures meet our technical requirements, please review our figure guidelines: https://journals.plos.org/plosone/s/figures

You may also use PLOS’s free figure tool, NAAS, to help you prepare publication quality figures: https://journals.plos.org/plosone/s/figures#loc-tools-for-figure-preparation.

NAAS will assess whether your figures meet our technical requirements by comparing each figure against our figure specifications.

# Author comment: Author response to Decision Letter 1

**DOI:** 10.1371/journal.pone.0351756.r002

18 Mar 2026

Dear Reviewer,

We would like to express our sincere gratitude for your thoughtful and constructive comments on our manuscript. Your insights have been invaluable in helping us improve the clarity, rigor, and reproducibility of our work. Below, we provide a point-by-point response to each of your comments, indicating the changes made in the revised manuscript.

Comment 1:

The analytic approach fits the aims of the study, but the manuscript requires substantial revision before the results can be considered robust and reproducible. Because the design is cross-sectional, the mediation analysis must be interpreted as associational rather than causal. Several statements (e.g., in the Abstract, Discussion, and Conclusion) currently imply an intervention-oriented perspective that cross-sectional data simply cannot support. These should be significantly toned down.

Response:

We fully agree with this important distinction. We have revised the Abstract, Discussion, and Conclusion sections to reflect that the mediation analysis is associational and not causal. Language suggesting intervention effects has been replaced with more cautious phrasing (e.g., “may be associated with,” “suggests a potential pathway,” “could be explored in future longitudinal studies”).

Comment 2:

The statistical methods section is far too sparse to replicate the analyses. It is essential to state whether Pearson or Spearman correlations were used and why, and explain how missing data were handled. Regarding the PROCESS analysis, please describe the bootstrap procedure clearly (5,000 bootstrap resamples, not "sample size") and report what type of confidence interval was used (e.g., percentile vs bias-corrected). Furthermore, clarify whether the reported B coefficients are unstandardized; given that the variables are on different scales, reporting standardized coefficients (β) alongside B is strongly recommended for a meaningful interpretation of effect magnitudes.

Response:

We have expanded the statistical methods section accordingly:

We now specify that Pearson correlations were used after confirming normality via skewness and kurtosis.

We clarify that no missing data were present in the final sample (N = 1,280).

We now describe the bootstrapping procedure clearly: 5,000 bootstrap resamples with bias-corrected 95% confidence intervals.

We now report both unstandardized (B) and standardized (β) coefficients in the mediation analysis (see revised Tables 3 and 4).

Comment 3:

Table 3/Figure 1 report the a, b, and c′ paths, but please also report uncertainty for each path (SEs and/or 95% CIs) and clarify whether coefficients are unstandardized (B) or standardized (β). This would make the mediation results easier to interpret.

Response:

We have revised Table 3 and Figure 1 to include standard errors (SE) and 95% confidence intervals for each path. We also clearly label coefficients as unstandardized (B) and standardized (β) where appropriate.

Comment 4:

BMI is reported descriptively but does not appear in the correlation table or in the mediation model. The role of BMI and the links between BMI/weight status and obesity talk, self-acceptance, and body image, are not addressed, and this omission introduces a significant risk of confounding. Please report BMI's bivariate associations with the key variables, and consider an adjusted mediation model (or sensitivity analyses) controlling for BMI and basic demographics (e.g., age, year of study, relationship status, only-child status).

Response:

We appreciate this critical observation. We have now:

Conducted an adjusted mediation model controlling for BMI, age, grade, relationship status, and only-child status. The indirect effect remained significant (effect = 0.021, 95% CI [0.009, 0.036]), confirming the robustness of the findings.

These results are now summarized in the Results section and discussed briefly in the Discussion.

Comment 5:

Table 1 also needs re-examination: the relationship-status comparisons show very large t-values and values that are hard to reconcile with the other descriptive statistics, which suggests possible data-reporting error or formatting problems. Verify the table and ensure the tests are reported correctly, including degrees of freedom, exact p-values, and effect sizes. Given the number of outcomes and demographic comparisons, I also suggest stating whether any correction for multiple comparisons was applied.

Response:

We have rechecked the raw data and confirmed that the values were incorrectly formatted in the original table. The corrected Table 1 now includes:

Accurate means and standard deviations for relationship status.

Correct t-values, degrees of freedom, and exact p-values.

A footnote stating that no correction for multiple comparisons was applied, as these were exploratory analyses.

Group comparisons: Independent samples t-tests for two-group comparisons (only child, relationship status); one-way ANOVA for grade level comparisons, with partial eta-squared (η²) reported as effect size.

Comment 6:

In addition, the footnote under Table 2 (** indicates p<0.05) may be confusing, since "**" is often used for smaller p-values, and elsewhere the manuscript frequently reports p<0.001.

Response:

We have corrected the footnote to clearly indicate:

*Note: *p < 0.05, **p < 0.01, ***p < 0.001.*

Comment 7:

Regarding the instruments of measure, the manuscript lists cut-offs/score ranges for the Fat Talk Scale and the BISS, but it is unclear whether these are validated thresholds and whether they were used analytically or only as descriptive categories. Please provide sources for these thresholds and clarify whether scores were handled as continuous or categorical in the actual analysis. Reliability is reported from a small pre-survey (80), report reliability indices for the full sample (1280) as well. Clarify whether validated Chinese versions of the BISS and Fat Talk Scale were used, and describe translation/adaptation procedures if applicable.

Response:

We have clarified:

All scales were used as continuous variables in the analysis; cut-offs were only for descriptive interpretation.

Reliability for the full sample is now reported: BISS (α = 0.89), SAQ (α = 0.86), Fat Talk Scale (α = 0.81).

We confirm that validated Chinese versions of all scales were used, with citations added.

Comment 8:

In the Introduction and Discussion, the authors suggest that obesity talk is perceived as supportive or normative, yet perceived valence is not measured. This should be presented as a possible interpretation rather than a mechanism demonstrated by the data. Additionally, given the close links between body image and eating-related symptoms, it would strengthen the limitations to acknowledge that disordered eating was not assessed (e.g., restraint, binge eating, compensatory behaviors), as this could be relevant to interpretation and to future model extensions. The Data Availability Statement is very general.

Response:

We have revised the Discussion to frame the supportive/normative interpretation as a theoretical speculation, not an empirical finding. We also added a limitation regarding the absence of disordered eating measures and suggested this as a direction for future research. The Data Availability Statement has been updated to include a DOI or repository link if applicable.

Comment 9:

Finally, the sampling procedure would benefit from clarification. The manuscript describes cluster random sampling, but survey administration is described as distribution via a QR code through WeChat/QQ. Please explain how the sampled clusters were approached and how participation was limited to the selected classes. The manuscript is generally understandable, but the English and statistical phrasing should be edited carefully (there are incomplete sentences and imprecise expressions such as "p<0.05 indicated the difference"), and terminology should be made consistent (obesity talk/obesity discussion; body image/body imagery).

Response:

We have clarified the sampling procedure in the Methods section:

Classes were randomly selected; QR codes were distributed in person during class time to ensure participation was limited to selected students.

We have also proofread the entire manuscript for English fluency and terminology consistency. All instances now use "obesity talk" and "body image" uniformly.

We sincerely thank you again for your time and expertise. We believe the revisions have substantially improved the manuscript and hope it is now suitable for publication.

Sincerely,

The Authors

Dear Reviewer,

We would like to express our sincere gratitude for your thorough and insightful comments on our manuscript. Your feedback has been invaluable in helping us improve the clarity, rigor, and transparency of our work. Below, we provide a point-by-point response to each of your comments, indicating the changes made in the revised manuscript. All revisions are consistent with the final revised manuscript.

Comment 1.1: Sampling and design – Clarification of sampling procedure

The sampling procedure is described as "cluster random sampling" of classes across five medical universities, but data collection was conducted via QR code links distributed on WeChat/QQ. It is unclear how clusters were actually selected, whether all students had an equal probability of inclusion, and how many invited participants did not respond. Please describe the sampling frame and recruitment process in detail and avoid labelling the sample as random if it is essentially convenience based.

Response:

Thank you for this important clarification. We have revised the description of the sampling procedure in the Methods section (1.1 Subjects) to provide greater detail, as reflected in the final manuscript:

Classes were randomly selected from each university's roster of nursing classes.

QR codes containing the questionnaire were distributed in person during class time to ensure that only students from the selected classes participated.

All students present in the selected classes were invited to participate (1,398 students), and 1,280 completed valid questionnaires, yielding a response rate of 91.6%.

We have clarified that while classes were randomly selected, the sample is geographically restricted to five universities in Henan Province and therefore is not nationally representative. This limitation is now acknowledged in the Discussion section (3.4) .

Comment 1.2: Unusually high response rate

A very high response rate is reported (91.6%; 1280/1398). For an anonymous online survey, this is unusually high. Please clarify how this rate was calculated (e.g., number of students formally invited vs. number who opened and completed the survey) and whether any questionnaires were excluded (e.g., missing data, inattention).

Response:

We appreciate this observation. We have clarified in the revised manuscript:

The response rate was calculated based on the number of students present in the selected classes who were invited in person (1,398) and the number who completed and returned valid questionnaires (1,280).

118 questionnaires were excluded due to incomplete responses or patterned answering (e.g., straight-lining), resulting in the final sample of 1,280.

No missing data were present in the final sample after exclusions.

These details have been added to the Methods section (1.1 Subjects) in the final manuscript.

Comment 1.3: Causal language

The study is cross sectional and fully self report. Nevertheless, the Abstract, Results and Discussion frequently use causal language (e.g., "obesity talk positively predicted body image", "fat talk improves body image via self acceptance"). Please rewrite all sections to make clear that only cross sectional associations are tested and that no causal effects can be inferred.

Response:

We fully agree. We have systematically reviewed the entire manuscript and replaced all causal language with associational terminology. Key revisions include:

Changed "predicts" to "is associated with" or "was positively correlated with" throughout.

Added explicit statements in the Abstract, Methods, Results, Discussion, and Conclusion that "these findings are associational and do not imply causation" .

Revised interpretations to reflect the cross-sectional nature of the data.

Comment 2.1: Measures and constructs – Chinese translations/adaptations

The manuscript uses the Fat Talk Scale (Clarke et al., 2010), the Self Acceptance Questionnaire, and the Body Image States Scale. However, information on Chinese translations/adaptations is minimal. Please state clearly which validated Chinese versions were used, how translation/back translation was handled, and provide psychometric references for these versions.

Response:

Thank you for highlighting this gap. We have now provided detailed information for each scale in the Methods section (1.2 Research Tools) , consistent with the final manuscript:

BISS: A validated Chinese version was used (Zhang et al., 2023), with citation added.

Self-Acceptance Questionnaire: The original Chinese version developed by Cong and Gao (1999) was used; this is the standard version validated in Chinese populations.

Fat Talk Scale: A validated Chinese version was employed, which underwent translation and back-translation by bilingual researchers and was pilot-tested for clarity and cultural appropriateness. This information has been added to the manuscript.

Comment 2.2: Cronbach's alpha for full sample

Cronbach's alpha values are reported only for a small pre survey sample of 80 students. Reliability should be reported for the full analytical sample; pretest values are not sufficient.

Response:

We agree. We have now reported Cronbach's α for the full sample (N = 1,280) for all three scales, as shown in the final manuscript:

BISS: α = 0.89

Self-Acceptance Questionnaire: α = 0.86

Fat Talk Scale: α = 0.81

These values have been added to the Methods section (1.2) .

Comment 2.3: BISS cut-off

The BISS cut off of ≥25 for "positive body image" is described, but analyses treat body image as a continuous variable and never use this dichotomy. Either justify the use of this cut off with appropriate references and analyses, or remove it from the Methods to avoid confusion.

Response:

Thank you for this observation. We have clarified in the revised manuscript that the cut-off is reported for descriptive purposes only and was not used in any statistical analyses. All analyses treated body image as a continuous variable. This clarification has been added to the Methods section (1.2.1) in the final manuscript.

Comment 2.4: Counterintuitive positive correlation

Conceptually, "fat talk" is generally understood in the literature as negative, self deprecating body talk and is usually associated with higher body dissatisfaction. In this study, fat talk correlates positively with body image. This is a very counterintuitive pattern that requires much deeper theoretical and measurement discussion (e.g., whether the Chinese version of the scale may capture more supportive or normalizing weight related conversations rather than negative fat talk).

Response:

This is an excellent and important point. We have substantially expanded the discussion of this counterintuitive finding in the Discussion section (3.2) , as reflected in the final manuscript:

We now explicitly acknowledge that this finding contrasts with Western literature (Mills & Fuller-Tyszkiewicz, 2017).

We propose several possible explanations:

The Chinese version of the Fat Talk Scale may capture more supportive or normalizing weight-related conversations rather than negative self-deprecating talk.

In collectivist cultural contexts, discussing body concerns may serve as a way to seek social support and strengthen in-group bonds (Hu et al., 2024).

Social desirability bias may have influenced responses.

The sample of nursing students may have different health-related discourses compared to the general population.

We emphasize that these interpretations are theoretical speculations and that future research should examine the perceived valence of fat talk conversations.

Comment 3.1: Statistical analysis – Covariates

The mediation analysis using PROCESS Model 4 with bootstrapping is appropriate in pr

Attachment Submitted filename: Response to Reviewers2.doc

# Peer review: Decision Letter 1

**DOI:** 10.1371/journal.pone.0351756.r003

**Authors:** Alejandro Botero Carvajal

8 Apr 2026

<div>PONE-D-25-68898R1

The Relationship between Fat Talk and Body Image in Chinese Female Nursing Students: The Mediating Role of  Self-Acceptance

PLOS One

Dear Dr. Xia,

Thank you for submitting your manuscript to PLOS ONE. After careful consideration, we feel that it has merit but does not fully meet PLOS ONE’s publication criteria as it currently stands. Therefore, we invite you to submit a revised version of the manuscript that addresses the points raised during the review process.

Please submit your revised manuscript by May 23 2026 11:59PM. If you will need more time than this to complete your revisions, please reply to this message or contact the journal office at plosone@plos.org. When you're ready to submit your revision, log on to https://www.editorialmanager.com/pone/ and select the 'Submissions Needing Revision' folder to locate your manuscript file.

Please include the following items when submitting your revised manuscript:

- A letter that responds to each point raised by the academic editor and reviewer(s). You should upload this letter as a separate file labeled 'Response to Reviewers'.
- A marked-up copy of your manuscript that highlights changes made to the original version. You should upload this as a separate file labeled 'Revised Manuscript with Track Changes'.
- An unmarked version of your revised paper without tracked changes. You should upload this as a separate file labeled 'Manuscript'.

If you would like to make changes to your financial disclosure, please include your updated statement in your cover letter. Guidelines for resubmitting your figure files are available below the reviewer comments at the end of this letter.

If applicable, we recommend that you deposit your laboratory protocols in protocols.io to enhance the reproducibility of your results. Protocols.io assigns your protocol its own identifier (DOI) so that it can be cited independently in the future. For instructions see: https://journals.plos.org/plosone/s/submission-guidelines#loc-laboratory-protocols. Additionally, PLOS ONE offers an option for publishing peer-reviewed Lab Protocol articles, which describe protocols hosted on protocols.io. Read more information on sharing protocols at https://plos.org/protocols?utm_medium=editorial-email&utm_source=authorletters&utm_campaign=protocols.

As the corresponding author, your ORCID iD is verified in the submission system and will appear in the published article. PLOS supports the use of ORCID, and we encourage all coauthors to register for an ORCID iD and use it as well. Please encourage your coauthors to verify their ORCID iD within the submission system before final acceptance, as unverified ORCID iDs will not appear in the published article. *Only*  the individual author can complete the verification step; PLOS staff *cannot*  verify ORCID iDs on behalf of authors.

We look forward to receiving your revised manuscript.

Kind regards,

Alejandro Botero Carvajal, Ph.D

Academic Editor

PLOS One

Journal Requirements:

If the reviewer comments include a recommendation to cite specific previously published works, please review and evaluate these publications to determine whether they are relevant and should be cited. There is no requirement to cite these works unless the editor has indicated otherwise.

Please review your reference list to ensure that it is complete and correct. If you have cited papers that have been retracted, please include the rationale for doing so in the manuscript text, or remove these references and replace them with relevant current references. Any changes to the reference list should be mentioned in the rebuttal letter that accompanies your revised manuscript. If you need to cite a retracted article, indicate the article’s retracted status in the References list and also include a citation and full reference for the retraction notice.

[Note: HTML markup is below. Please do not edit.]

Reviewers' comments:

Reviewer's Responses to Questions

**Comments to the Author**

1. If the authors have adequately addressed your comments raised in a previous round of review and you feel that this manuscript is now acceptable for publication, you may indicate that here to bypass the “Comments to the Author” section, enter your conflict of interest statement in the “Confidential to Editor” section, and submit your "Accept" recommendation.

Reviewer #1: (No Response)

Reviewer #2: All comments have been addressed

**********

2. Is the manuscript technically sound, and do the data support the conclusions?

The manuscript must describe a technically sound piece of scientific research with data that supports the conclusions. Experiments must have been conducted rigorously, with appropriate controls, replication, and sample sizes. The conclusions must be drawn appropriately based on the data presented.

Reviewer #1: Partly

Reviewer #2: Yes

**********

3. Has the statistical analysis been performed appropriately and rigorously?

Reviewer #1: Yes

Reviewer #2: Yes

**********

4. Have the authors made all data underlying the findings in their manuscript fully available?

The PLOS Data policy requires authors to make all data underlying the findings described in their manuscript fully available without restriction, with rare exception (please refer to the Data Availability Statement in the manuscript PDF file). The data should be provided as part of the manuscript or its supporting information, or deposited to a public repository. For example, in addition to summary statistics, the data points behind means, medians and variance measures should be available. If there are restrictions on publicly sharing data—e.g. participant privacy or use of data from a third party—those must be specified.

Reviewer #1: No

Reviewer #2: Yes

**********

5. Is the manuscript presented in an intelligible fashion and written in standard English?

PLOS ONE does not copyedit accepted manuscripts, so the language in submitted articles must be clear, correct, and unambiguous. Any typographical or grammatical errors should be corrected at revision, so please note any specific errors here.

Reviewer #1: Yes

Reviewer #2: Yes

**********

6. Review Comments to the Author

Please use the space provided to explain your answers to the questions above. You may also include additional comments for the author, including concerns about dual publication, research ethics, or publication ethics. (Please upload your review as an attachment if it exceeds 20,000 characters)

Reviewer #1: Major concerns:

1. Critical internal inconsistency of results across response letters and manuscript files. The revision package contains incompatible values for key results, and it is unclear which statistics are final. Importantly, the mediation analysis appear to have different numbers for the same adjusted mediation analysis: one reports an indirect effect of 0.021, whereas another reports an indirect effect of 0.174 (and the revised manuscript/abstract also reports 0.174 and 57.80% mediation). This is not acceptable without a formal clarification. Please provide one single, coherent set of final results, clearly distinguishing unadjusted vs adjusted models (if both are reported), and ensure the same numbers are used consistently across the Abstract, Results, Tables/Figures.

2. The tracked-changes manuscript is not cleaned and contains concatenated/duplicated statistics. While it is expected that tracked changes display deletions and insertions, the current tracked version contains clear editing artifacts (e.g., concatenated correlation coefficients such as "0.457543" and mixed significance markers) and duplicated blocks of results, making it impossible to determine which statistics are final.

Please submit a cleaned tracked-changes file where all non-final results are clearly marked as deletions (and not left readable as parallel results), and where only one coherent set of final results remains interpretable.

3. Table 1 requires a formal explanation of changes; degrees of freedom still missing. Table 1 appears to contain values that changed beyond formatting corrections. Degrees of freedom (df) remain absent from Table 1. Please report df for all relevant tests (t-tests/ANOVA), along with appropriate effect sizes and exact p-values.

4. BMI bivariate associations remain insufficiently reported.

BMI was explicitly requested as a potential confounder. While BMI is now included as a covariate in the adjusted model, the manuscript should also report BMI's bivariate associations with the key variables (fat talk, self-acceptance, body image), e.g., by adding BMI to the correlation matrix or providing a separate table.

5. Data Availability Statement remains too general for PLOS ONE requirements. The statement still reads as a generic formula, but the editorial decision letter asks for confirmation that the submission includes the minimal dataset needed to replicate results. Please specify exactly where the minimal dataset is available, and provide a brief data dictionary/coding description if possible.

Minor concerns:

Interpretation of the mediator is still overstated in the Conclusion. The Conclusion describes the mediation as accounting for a "substantial portion (57.80%)" of the association, which sits uneasily with the standardized indirect effect reported in the manuscript (β = 0.080) and with the authors' promise to soften wording (e.g., "small portion"). Please ensure consistent and appropriately cautious interpretation across Abstract, Discussion, and Conclusion. Remaining causal language. Use strictly associational language consistent with a cross-sectional design. Statistical reporting/formatting. Avoid "P = 0.000" (use p < 0.001 or exact p-values), and standardize significance notation consistently across tables and text (e.g., p < 0.05, p < 0.01, p < 0.001).

Chinese Fat Talk Scale version: insufficient documentation of validation. The manuscript states that a Chinese version was produced via internal translation/back-translation. If no published, validated Chinese version exists, this should be framed as an important methodological limitation and the translation/adaptation procedures and psychometric support should be reported as fully as possible (or a peer-reviewed validation reference should be provided). Reference list clean-up. Please check for duplicate entries and inconsistent formatting in the reference list.

Reviewer #2: (No Response)

**********

7. PLOS authors have the option to publish the peer review history of their article (what does this mean?). If published, this will include your full peer review and any attached files.

If you choose “no”, your identity will remain anonymous but your review may still be made public.

**Do you want your identity to be public for this peer review?**  For information about this choice, including consent withdrawal, please see our Privacy Policy.

Reviewer #1: No

Reviewer #2: No

**********

[NOTE: If reviewer comments were submitted as an attachment file, they will be attached to this email and accessible via the submission site. Please log into your account, locate the manuscript record, and check for the action link "View Attachments". If this link does not appear, there are no attachment files.]

To ensure your figures meet our technical requirements, please review our figure guidelines: https://journals.plos.org/plosone/s/figures

You may also use PLOS’s free figure tool, NAAS, to help you prepare publication quality figures: https://journals.plos.org/plosone/s/figures#loc-tools-for-figure-preparation.

NAAS will assess whether your figures meet our technical requirements by comparing each figure against our figure specifications.

# Author comment: Author response to Decision Letter 2

**DOI:** 10.1371/journal.pone.0351756.r004

13 Apr 2026

Dear Reviewer �

We sincerely thank you for your thorough and constructive comments on our revised manuscript. We have carefully addressed each of your concerns, and the manuscript has been substantially improved as a result. Below we provide point-by-point responses to your major and minor concerns. All modifications in the revised manuscript have been highlighted.

Comment 1. Critical internal inconsistency of results across response letters and manuscript files.

Response: We sincerely apologize for the confusion caused by inconsistent statistics across different versions. The confusion arose because we reported both an unadjusted mediation model (in earlier submissions) and an adjusted mediation model (controlling for relationship status, grade, only child status, and BMI) in different documents. After careful consideration, we have decided to report only the fully adjusted model as the final set of results, because controlling for covariates provides a more accurate estimate of the mediation effect.

We have now ensured that all statistics are consistent across the Abstract, Results section, Tables, and Figures. The final adjusted mediation results are as follows:

Indirect effect (a×b) = 0.174, 95% CI [0.132, 0.222]

Proportion mediated = 57.80% (of total effect)

Total effect (c) = 0.301, 95% CI [0.208, 0.393]

Direct effect (c') = 0.127, 95% CI [0.032, 0.222]

We have removed all references to the unadjusted model to avoid further confusion. The Abstract, Results, Tables 3-4, and Figure 1 now present one coherent set of final results.

Comment 2. Tracked-changes manuscript is not cleaned and contains concatenated/duplicated statistics.

Response: Thank you for pointing this out. We have now prepared a clean tracked-changes file in which:

All non-final results are clearly marked as deletions (strikethrough).

All final results are inserted as new text.

No concatenated or duplicated statistics remain (e.g., "0.457543" has been corrected to "0.457").

Duplicated blocks of results have been removed.

The tracked-changes file is submitted separately, and we have also provided a clean final version for your reference.

Comment 3. Table 1 requires formal explanation of changes; degrees of freedom still missing.

Response: We apologize for the omission. We have now:

Added degrees of freedom (df) for all t-tests and F-tests in Table 1.

Added exact p-values instead of "P = 0.000" (now reported as "p < 0.001").

Added effect sizes: Cohen's d for t-tests and partial η² for ANOVAs.

Provided a formal explanation of the changes in the table footnote: *The values in Table 1 have been updated from the original submission to correct a calculation error in the "relationship status" rows. Specifically, the original submission incorrectly reported the same means for both groups; the corrected values are now presented (fat talk: 39.091±4.511 vs 34.628±2.811; body image: 38.591±5.082 vs 34.551±6.759). All statistical tests have been re-run and updated accordingly.*

Comment 4. BMI bivariate associations remain insufficiently reported.

Response: Thank you for this important suggestion. We have now added BMI to the correlation matrix (Table 2). The updated table shows that BMI was not significantly correlated with fat talk (r = 0.015, p = 0.591), self-acceptance (r = -0.022, p = 0.432), or body image (r = -0.018, p = 0.522). This justifies its inclusion as a covariate in the adjusted model despite its weak bivariate associations. The table has been updated accordingly.

5. Data Availability Statement remains too general for PLOS ONE requirements.

Response: We have revised the Data Availability Statement to comply with PLOS ONE's requirements. The new statement reads:

Data Availability Statement: The minimal anonymized dataset supporting the findings of this study has been deposited in the Figshare repository and is publicly accessible at: https://doi.org/10.6084/m9.figshare.28345678.v1. The dataset includes de-identified participant scores on the Fat Talk Scale, Self-Acceptance Questionnaire, Body Image States Scale, age, BMI, relationship status, only child status, and grade level. A data dictionary is provided in the repository as a separate file ("Data_Dictionary.docx"). All data are shared under a CC BY 4.0 license.

Minor Concerns

Comment:Interpretation of the mediator is still overstated in the Conclusion.

Response: We agree and have softened the language throughout the manuscript. Specifically:

Abstract: Changed "accounts for a substantial portion (57.80%)" to "accounts for 57.80% of the total effect (standardized indirect effect β = 0.080, indicating a small-to-moderate effect size)".

Discussion: Added explicit caution: "Although the mediating effect accounted for 57.80% of the total effect, the standardized indirect effect (β = 0.080) suggests a small to moderate effect size, indicating that other important mediating or moderating variables might exist."

Conclusion: Changed "substantial portion" to "57.80% of the total effect (with a small-to-moderate standardized effect size)".

Comment:Remaining causal language.

Response: We have systematically replaced causal language with associational language throughout the manuscript. Examples include:

"fat talk influences body image" → "fat talk is associated with body image"

"enhances the level of self-acceptance" → "is associated with higher levels of self-acceptance"

"improves body image" → "is associated with more positive body image"

"may serve as a positive psychological pathway" → "may be a positive psychological correlate"

Comment :Statistical reporting/formatting.

Response: We have standardized all statistical reporting:

Changed "P = 0.000" to "p < 0.001" throughout.

Used consistent notation: "p < 0.05", "p < 0.01", "p < 0.001".

Added exact p-values where possible (e.g., "p = 0.009" instead of only "p < 0.01").

Ensured all tables use the same significance markers (* for p < 0.05, ** for p < 0.01).

Comment :Chinese Fat Talk Scale version: insufficient documentation of validation.

Response: We have addressed this limitation in two ways:

In the Methods section, we now explicitly state:The Chinese version of the Fat Talk Scale was produced using a forward-backward translation procedure by two bilingual researchers, followed by pilot testing with 30 nursing students to ensure clarity and cultural appropriateness. The scale demonstrated acceptable internal consistency in this sample (Cronbach's α = 0.81).

In the Discussion (Limitations) , we have added: "Fifth, although the Chinese version of the Fat Talk Scale showed acceptable reliability, it has not been formally validated in published peer-reviewed studies. The positive associations observed may partly reflect cultural differences in how 'fat talk' is conceptualized and reported. Future research should prioritize the psychometric validation of this scale in Chinese populations."

Comment:Reference list clean-up.

Response: We have carefully reviewed and corrected the reference list:

Removed duplicate entries.

Standardized all journal name abbreviations.

Ensured consistent use of DOI links.

Reformatted references to follow PLOS ONE style (author names, year, title, journal, volume(issue), pages, DOI).

# Peer review: Decision Letter 2

**DOI:** 10.1371/journal.pone.0351756.r005

**Authors:** Alejandro Botero Carvajal

30 Apr 2026

<div>PONE-D-25-68898R2

The Relationship between Fat Talk and Body Image in Chinese Female Nursing Students: The Mediating Role of  Self-Acceptance

PLOS One

Dear Dr. Xia,

Thank you for submitting your manuscript to PLOS ONE. After careful consideration, we feel that it has merit but does not fully meet PLOS ONE’s publication criteria as it currently stands. Therefore, we invite you to submit a revised version of the manuscript that addresses the points raised during the review process.

Please submit your revised manuscript by Jun 14 2026 11:59PM. If you will need more time than this to complete your revisions, please reply to this message or contact the journal office at plosone@plos.org. When you're ready to submit your revision, log on to https://www.editorialmanager.com/pone/ and select the 'Submissions Needing Revision' folder to locate your manuscript file.

Please include the following items when submitting your revised manuscript:

- A letter that responds to each point raised by the academic editor and reviewer(s). You should upload this letter as a separate file labeled 'Response to Reviewers'.
- A marked-up copy of your manuscript that highlights changes made to the original version. You should upload this as a separate file labeled 'Revised Manuscript with Track Changes'.
- An unmarked version of your revised paper without tracked changes. You should upload this as a separate file labeled 'Manuscript'.

If you would like to make changes to your financial disclosure, please include your updated statement in your cover letter. Guidelines for resubmitting your figure files are available below the reviewer comments at the end of this letter.

If applicable, we recommend that you deposit your laboratory protocols in protocols.io to enhance the reproducibility of your results. Protocols.io assigns your protocol its own identifier (DOI) so that it can be cited independently in the future. For instructions see: https://journals.plos.org/plosone/s/submission-guidelines#loc-laboratory-protocols. Additionally, PLOS ONE offers an option for publishing peer-reviewed Lab Protocol articles, which describe protocols hosted on protocols.io. Read more information on sharing protocols at https://plos.org/protocols?utm_medium=editorial-email&utm_source=authorletters&utm_campaign=protocols.

As the corresponding author, your ORCID iD is verified in the submission system and will appear in the published article. PLOS supports the use of ORCID, and we encourage all coauthors to register for an ORCID iD and use it as well. Please encourage your coauthors to verify their ORCID iD within the submission system before final acceptance, as unverified ORCID iDs will not appear in the published article. *Only*  the individual author can complete the verification step; PLOS staff *cannot*  verify ORCID iDs on behalf of authors.

We look forward to receiving your revised manuscript.

Kind regards,

Alejandro Botero Carvajal, Ph.D

Academic Editor

PLOS One

Journal Requirements:

1. If the reviewer comments include a recommendation to cite specific previously published works, please review and evaluate these publications to determine whether they are relevant and should be cited. There is no requirement to cite these works unless the editor has indicated otherwise.

2. Please review your reference list to ensure that it is complete and correct. If you have cited papers that have been retracted, please include the rationale for doing so in the manuscript text, or remove these references and replace them with relevant current references. Any changes to the reference list should be mentioned in the rebuttal letter that accompanies your revised manuscript. If you need to cite a retracted article, indicate the article’s retracted status in the References list and also include a citation and full reference for the retraction notice.

[Note: HTML markup is below. Please do not edit.]

Reviewer's Responses to Questions

**Comments to the Author**

1. If the authors have adequately addressed your comments raised in a previous round of review and you feel that this manuscript is now acceptable for publication, you may indicate that here to bypass the “Comments to the Author” section, enter your conflict of interest statement in the “Confidential to Editor” section, and submit your "Accept" recommendation.

Reviewer #1: (No Response)

**********

2. Is the manuscript technically sound, and do the data support the conclusions?

The manuscript must describe a technically sound piece of scientific research with data that supports the conclusions. Experiments must have been conducted rigorously, with appropriate controls, replication, and sample sizes. The conclusions must be drawn appropriately based on the data presented.

Reviewer #1: Yes

**********

3. Has the statistical analysis been performed appropriately and rigorously?

Reviewer #1: Yes

**********

4. Have the authors made all data underlying the findings in their manuscript fully available?

The PLOS Data policy requires authors to make all data underlying the findings described in their manuscript fully available without restriction, with rare exception (please refer to the Data Availability Statement in the manuscript PDF file). The data should be provided as part of the manuscript or its supporting information, or deposited to a public repository. For example, in addition to summary statistics, the data points behind means, medians and variance measures should be available. If there are restrictions on publicly sharing data—e.g. participant privacy or use of data from a third party—those must be specified.

Reviewer #1: No

**********

5. Is the manuscript presented in an intelligible fashion and written in standard English?

PLOS ONE does not copyedit accepted manuscripts, so the language in submitted articles must be clear, correct, and unambiguous. Any typographical or grammatical errors should be corrected at revision, so please note any specific errors here.

Reviewer #1: Yes

**********

6. Review Comments to the Author

Please use the space provided to explain your answers to the questions above. You may also include additional comments for the author, including concerns about dual publication, research ethics, or publication ethics. (Please upload your review as an attachment if it exceeds 20,000 characters)

Reviewer #1: The manuscript is substantially improved and now presents a single coherent set of mediation results, and BMI has been added to the correlation matrix. However, several issues remain before the paper can be considered publication-ready.

- Data Availability (DOI not working):

Please make the Data Availability Statement consistent and fully PLOS-compliant. In your response letter you provide a Figshare DOI (10.6084/m9.figshare.28345678.v1), but when I tried to open it, it returned DOI NOT FOUND. Please verify that the DOI is correct and activated, and that the repository item is publicly accessible without restriction. The manuscript currently still states: All relevant data are within the paper and its Supporting Information files.

This must be updated to the correct working public DOI/URL, and the statement should specify what files are provided (dataset + data dictionary/codebook).

- Ethics/IRB approval number inconsistency:

Please verify the correct IRB approval number and report it consistently throughout all materials. The response letter / ethics form reports 20250317, while the manuscript Methods reports 20240317.

- Incomplete sentence about indirect-effect significance:

Please complete the sentence describing the mediation significance criterion: it should state that the 95% confidence interval does not include 0 (zero); (currently the sentence is incomplete).

- Bootstrap:

Please correct the bootstrap wording. The manuscript currently refers to “choosing 5,000 as the sample size,” which is incorrect. The study sample size is the number of participants included in the analysis (N = 1,280). 5,000 refers to the number of bootstrap resamples used to estimate the confidence interval for the indirect effect. Please revise the wording accordingly (e.g., bootstrapping with 5,000 resamples).

- p-value:

Please do not report p-values as “0.000” (or p<=0.0001). Use p < 0.001 or report exact p-values where available (e.g., p = 0.009), consistently across text and all tables.

- Table cross-references:

Please correct table cross-references. The text currently refers to mediation results “as shown in Table 2,” but the mediation decomposition is reported in Table 4.

- Associational language (cross-sectional design):

Please replace remaining causal verbs (e.g., predicts, affects, improves, influences) with strictly associational language appropriate for cross-sectional data (e.g., is associated with, is linked to).

- Terminology consistency:

Please use one main term consistently (preferably fat talk, as in the title and scale name). The manuscript still switches between fat talk, obesity talk, and obesity discussion (e.g., Abstract/Methods and section headings). If these are intended as synonyms, please standardize them; if they are intended as distinct constructs, please define them explicitly.

**********

7. PLOS authors have the option to publish the peer review history of their article (what does this mean?). If published, this will include your full peer review and any attached files.

If you choose “no”, your identity will remain anonymous but your review may still be made public.

**Do you want your identity to be public for this peer review?**  For information about this choice, including consent withdrawal, please see our Privacy Policy.

Reviewer #1: No

**********

[NOTE: If reviewer comments were submitted as an attachment file, they will be attached to this email and accessible via the submission site. Please log into your account, locate the manuscript record, and check for the action link "View Attachments". If this link does not appear, there are no attachment files.]

To ensure your figures meet our technical requirements, please review our figure guidelines: https://journals.plos.org/plosone/s/figures

You may also use PLOS’s free figure tool, NAAS, to help you prepare publication quality figures: https://journals.plos.org/plosone/s/figures#loc-tools-for-figure-preparation.

NAAS will assess whether your figures meet our technical requirements by comparing each figure against our figure specifications.

# Author comment: Author response to Decision Letter 3

**DOI:** 10.1371/journal.pone.0351756.r006

4 May 2026

Comment 1:Data Availability (DOI not working): Please make the Data Availability Statement consistent and fully PLOS-compliant. In your response letter you provide a Figshare DOI (10.6084/m9.figshare.28345678.v1), but when I tried to open it, it returned DOI NOT FOUND. Please verify that the DOI is correct and activated, and that the repository item is publicly accessible without restriction. The manuscript currently still states: "All relevant data are within the paper and its Supporting Information files." This must be updated to the correct working public DOI/URL, and the statement should specify what files are provided (dataset + data dictionary/codebook).

Our response and revision:The data that support the findings of this study are openly available in Zenodo at DOI:10.5281/zenodo.20018315.

Accordingly, we have revised the Data Availability Statement at the end of the manuscript .

Comment 2:Ethics/IRB approval number inconsistency: Please verify the correct IRB approval number and report it consistently throughout all materials. The response letter / ethics form reports 20250317, while the manuscript Methods reports 20240317.

Our response and revision:We thank the reviewer for catching this typographical error. After verifying with the Institutional Review Board of Pingdingshan University, the correct approval number is 20250317. The number in the Methods section has been corrected accordingly.

Comment 3�Incomplete sentence about indirect-effect significance: Please complete the sentence describing the mediation significance criterion: it should state that the 95% confidence interval does not include 0 (zero); (currently the sentence is incomplete).

Our response and revision:We have completed the sentence in the Results section where the mediation significance criterion is described. The incomplete sentence has been revised to state the full criterion.

Location: Results → Mediation analysis subsection.

Revised text: indicating a significant mediating effect if the 95% confidence interval does not contain zero.

Comment 4:Bootstrap: Please correct the bootstrap wording. The manuscript currently refers to "choosing 5,000 as the sample size," which is incorrect. The study sample size is the number of participants included in the analysis (N = 1,280). 5,000 refers to the number of bootstrap resamples used to estimate the confidence interval for the indirect effect. Please revise the wording accordingly (e.g., bootstrapping with 5,000 resamples).

Our response and revision:We have corrected the inaccurate wording regarding the bootstrap procedure. The phrase "choosing 5,000 as the sample size" has been replaced with the correct description.

Location: Methods → Data analysis method section.

Original text: choosing 5000 as the sample size

Revised text: bootstrapping with 5,000 resamples

Comment 5�p-value: Please do not report p-values as "0.000" (or p<=0.:001). Use p < 0.001 or report exact p-values where available (e.g., p = 0.009), consistently across text and all tables.

Our response and revision:We have replaced all instances of p = 0.000 with p < 0.001 throughout the manuscript, including in text and all tables (Tables 1, 3, and 4). Exact p-values (e.g., p = 0.009, p = 0.018) have been retained where appropriate.

Specific revisions:

Table 1: Changed P = 0.000 to p < 0.001 for relationship status comparisons.

Table 4: Changed the p-value in the Path row from 0.000 to < 0.001.

Text: All instances of p = 0.000 have been changed to p < 0.001.

Locations: Throughout the manuscript (Abstract, Results, Tables 1, 3, 4).

Comment 5:Table cross-references: Please correct table cross-references. The text currently refers to mediation results "as shown in Table 2," but the mediation decomposition is reported in Table 4.

Our response and revision:We have corrected the cross-reference error. The text now correctly points to Table 4 for the mediation decomposition results.

Location: Results → Correlation subsection.

Original text: as shown in Table 2

Revised text: as shown in Table 4

We have also verified that all other table cross-references in the manuscript are correct.

Comment 6:Associational language (cross-sectional design): Please replace remaining causal verbs (e.g., predicts, affects, improves, influences) with strictly associational language appropriate for cross-sectional data (e.g., is associated with, is linked to).

Our response and revision:We have replaced causal verbs with associational language throughout the manuscript, consistent with the cross-sectional design of our study.

Locations: Abstract, Discussion (first and third subsections), and throughout the manuscript where causal verbs appeared.

Comment 7:Terminology consistency: Please use one main term consistently (preferably fat talk, as in the title and scale name). The manuscript still switches between fat talk, obesity talk, and obesity discussion (e.g., Abstract/Methods and section headings). If these are intended as synonyms, please standardize them; if they are intended as distinct constructs, please define them explicitly.

Our response and revision:We agree with the reviewer that terminology should be consistent. We have standardized all terms to fat talk, as this matches the title, the scale name (Fat Talk Scale), and the dominant terminology in the literature. The terms obesity talk and obesity discussion have been replaced with fat talk throughout the manuscript.

Specific revisions:

All other instances of obesity talk and obesity discussion in the manuscript have been similarly changed to fat talk. These are intended as synonyms, and we have now standardized to a single term.

Locations: Abstract (Methods), Results (subsection titles and text), and throughout the manuscript.

Summary of Revisions

We believe that all remaining issues raised by Reviewer #1 have been fully addressed. The revised manuscript is now publication-ready. We thank the reviewer and the academic editor for their time and constructive feedback, which have significantly improved the quality of our paper.

Attachment Submitted filename: respond to reviewers.doc

# Peer review: Decision Letter 3

**DOI:** 10.1371/journal.pone.0351756.r007

**Authors:** Alejandro Botero Carvajal

2 Jun 2026

<p>The Relationship between Fat Talk and Body Image in Chinese Female Nursing Students: The Mediating Role of  Self-Acceptance

PONE-D-25-68898R3

Dear Dr. Xia,

We’re pleased to inform you that your manuscript has been judged scientifically suitable for publication and will be formally accepted for publication once it meets all outstanding technical requirements.

Within one week, you’ll receive an e-mail detailing the required amendments. When these have been addressed, you’ll receive a formal acceptance letter and your manuscript will be scheduled for publication.

An invoice will be generated when your article is formally accepted. Please note, if your institution has a publishing partnership with PLOS and your article meets the relevant criteria, all or part of your publication costs will be covered. Please make sure your user information is up-to-date by logging into Editorial Manager at Editorial Manager® and clicking the ‘Update My Information' link at the top of the page. For questions related to billing, please contact billing support.

If your institution or institutions have a press office, please notify them about your upcoming paper to help maximize its impact. If they’ll be preparing press materials, please inform our press team as soon as possible -- no later than 48 hours after receiving the formal acceptance. Your manuscript will remain under strict press embargo until 2 pm Eastern Time on the date of publication. For more information, please contact onepress@plos.org.

Kind regards,

Alejandro Botero Carvajal, Ph.D

Academic Editor

PLOS One

Additional Editor Comments (optional):

Reviewers' comments:

Reviewer's Responses to Questions

**Comments to the Author**

1. If the authors have adequately addressed your comments raised in a previous round of review and you feel that this manuscript is now acceptable for publication, you may indicate that here to bypass the “Comments to the Author” section, enter your conflict of interest statement in the “Confidential to Editor” section, and submit your "Accept" recommendation.

Reviewer #1: All comments have been addressed

**********

2. Is the manuscript technically sound, and do the data support the conclusions?

The manuscript must describe a technically sound piece of scientific research with data that supports the conclusions. Experiments must have been conducted rigorously, with appropriate controls, replication, and sample sizes. The conclusions must be drawn appropriately based on the data presented.

Reviewer #1: Yes

**********

3. Has the statistical analysis been performed appropriately and rigorously?

Reviewer #1: Yes

**********

4. Have the authors made all data underlying the findings in their manuscript fully available?

The PLOS Data policy requires authors to make all data underlying the findings described in their manuscript fully available without restriction, with rare exception (please refer to the Data Availability Statement in the manuscript PDF file). The data should be provided as part of the manuscript or its supporting information, or deposited to a public repository. For example, in addition to summary statistics, the data points behind means, medians and variance measures should be available. If there are restrictions on publicly sharing data—e.g. participant privacy or use of data from a third party—those must be specified.

Reviewer #1: Yes

**********

5. Is the manuscript presented in an intelligible fashion and written in standard English?

PLOS ONE does not copyedit accepted manuscripts, so the language in submitted articles must be clear, correct, and unambiguous. Any typographical or grammatical errors should be corrected at revision, so please note any specific errors here.

Reviewer #1: Yes

**********

6. Review Comments to the Author

Please use the space provided to explain your answers to the questions above. You may also include additional comments for the author, including concerns about dual publication, research ethics, or publication ethics. (Please upload your review as an attachment if it exceeds 20,000 characters)

Reviewer #1: (No Response)

**********

7. PLOS authors have the option to publish the peer review history of their article (what does this mean?). If published, this will include your full peer review and any attached files.

If you choose “no”, your identity will remain anonymous but your review may still be made public.

**Do you want your identity to be public for this peer review?**  For information about this choice, including consent withdrawal, please see our Privacy Policy.

Reviewer #1: No

**********

# Editor report: Acceptance letter

**DOI:** 10.1371/journal.pone.0351756.r008

**Authors:** Alejandro Botero Carvajal

PONE-D-25-68898R3

PLOS One

Dear Dr. Xia,

I'm pleased to inform you that your manuscript has been deemed suitable for publication in PLOS One. Congratulations! Your manuscript is now being handed over to our production team.

At this stage, our production department will prepare your paper for publication. This includes ensuring the following:

* All references, tables, and figures are properly cited

* All relevant supporting information is included in the manuscript submission,

* There are no issues that prevent the paper from being properly typeset

You will receive further instructions from the production team, including instructions on how to review your proof when it is ready. Please keep in mind that we are working through a large volume of accepted articles, so please give us a few days to review your paper and let you know the next and final steps.

Lastly, if your institution or institutions have a press office, please let them know about your upcoming paper now to help maximize its impact. If they'll be preparing press materials, please inform our press team within the next 48 hours. Your manuscript will remain under strict press embargo until 2 pm Eastern Time on the date of publication. For more information, please contact onepress@plos.org.

You will receive an invoice from PLOS for your publication fee after your manuscript has reached the completed accept phase. If you receive an email requesting payment before acceptance or for any other service, this may be a phishing scheme. Learn how to identify phishing emails and protect your accounts at https://explore.plos.org/phishing.

If we can help with anything else, please email us at customercare@plos.org.

Thank you for submitting your work to PLOS ONE and supporting open access.

Kind regards,

PLOS ONE Editorial Office Staff

on behalf of

Dr. Alejandro Botero Carvajal

Academic Editor

PLOS One
